# Protective effect of zinc gluconate on intestinal mucosal barrier injury in antibiotics and LPS-induced mice

**DOI:** 10.3389/fmicb.2024.1407091

**Published:** 2024-05-23

**Authors:** Yongcai Wang, Juan Xiao, Sumei Wei, Ying Su, Xia Yang, Shiqi Su, Liancheng Lan, Xiuqi Chen, Ting Huang, Qingwen Shan

**Affiliations:** ^1^Department of Pediatrics, The First Affiliated Hospital of Guangxi Medical University, Nanning, China; ^2^Dazhou Central Hospital, Dazhou, China; ^3^Guangxi Key Laboratory of Aquatic Genetic Breeding and Healthy Aquaculture, Guangxi Academy of Fishery Sciences, Nanning, China

**Keywords:** zinc gluconate, intestinal mucosal barrier, gut microbiome, antibiotics, LPS, TLR4/NF-κB, tight junction, 16S rRNA

## Abstract

**Objective:**

The aim of the study is to investigate the function and mechanism of Zinc Gluconate (ZG) on intestinal mucosal barrier damage in antibiotics and Lipopolysaccharide (LPS)-induced mice.

**Methods:**

We established a composite mouse model by inducing intestinal mucosal barrier damage using antibiotics and LPS. The animals were divided into five groups: Control (normal and model) and experimental (low, medium, and high-dose ZG treatments). We evaluated the intestinal mucosal barrier using various methods, including monitoring body weight and fecal changes, assessing pathological damage and ultrastructure of the mouse ileum, analyzing expression levels of tight junction (TJ)-related proteins and genes, confirming the TLR4/NF-κB signaling pathway, and examining the structure of the intestinal flora.

**Results:**

In mice, the dual induction of antibiotics and LPS led to weight loss, fecal abnormalities, disruption of ileocecal mucosal structure, increased intestinal barrier permeability, and disorganization of the microbiota structure. ZG restored body weight, alleviated diarrheal symptoms and pathological damage, and maintained the structural integrity of intestinal epithelial cells (IECs). Additionally, ZG reduced intestinal mucosal permeability by upregulating TJ-associated proteins (ZO-1, Occludin, Claudin-1, and JAM-A) and downregulating MLCK, thereby repairing intestinal mucosal barrier damage induced by dual induction of antibiotics and LPS. Moreover, ZG suppressed the TLR4/NF-κB signaling pathway, demonstrating anti-inflammatory properties and preserving barrier integrity. Furthermore, ZG restored gut microbiota diversity and richness, evidenced by increased Shannon and Observed features indices, and decreased Simpson’s index. ZG also modulated the relative abundance of beneficial human gut bacteria (*Bacteroidetes*, *Firmicutes*, *Verrucomicrobia*, *Parabacteroides*, *Lactobacillus*, and *Akkermansia*) and harmful bacteria (*Proteobacteria* and *Enterobacter*), repairing the damage induced by dual administration of antibiotics and LPS.

**Conclusion:**

ZG attenuates the dual induction of antibiotics and LPS-induced intestinal barrier damage and also protects the intestinal barrier function in mice.

## 1 Introduction

Intestinal mucosal barrier integrity is essential for maintaining intestinal health and preventing related diseases. However, in Antibiotic-Associated Colitis (AAC), the antibiotic-induced microecological imbalance not only causes direct damage to the intestinal mucosa, but also impairs the intestinal barrier function, increasing its vulnerability to harmful external stimuli (Xu et al., 2023). In this study, we aim to investigate the function and mechanism of Zinc Gluconate (ZG) on intestinal mucosal barrier damage using a composite model of the dual induction of antibiotics and Lipopolysaccharide (LPS)-induced intestinal mucosal barrier injury. We also examined the effect of ZG on intestinal flora.

Antibiotics significantly impact the microbial population in the gut, reducing the abundance of beneficial bacterial, whereas increasing the abundance of harmful bacteria. The microecological imbalance disrupts the mucosal barrier, as evidenced by increased mucosal barrier permeability, aberrant expression of mucosal barrier proteins, and mucosal barrier cell apoptosis. Furthermore, the disruption lowers the intestinal mucosal barrier resistance to external pathogenic factors, creating a potential pathway for AAC (Kaminsky et al., 2021; Xu et al., 2023). Antibiotics induce a microbial imbalance, which, in turn, disrupts the mechanical barrier, significantly lowering the gut mucosa’s resistance to reinfection by Gram-negative bacteria. Specifically, antibiotic-induced changes in the microbial community severely impaired the biological barrier function, resulting in reduced mucus production and secretion of antimicrobial peptides (Kwon et al., 2022; Li X. et al., 2023). This impact led to an increased susceptibility to reinfection with Gram-negative bacteria, offering novel insights into the mechanisms underlying antibiotic-induced mucosal barrier damage. Furthermore, LPS exposure could cause increased permeability and disruption of the integrity of the gastrointestinal mucosal epithelial Tight Junction (TJ). This process allows endotoxins to infiltrate the gut via paracellular osmosis, causing endotoxin translocation and aberrant cytokine expression, thereby worsening intestinal inflammation and injury (Zhang and Li, 2012; Zhao et al., 2023). Therefore, antibiotic-induced microecological dysregulation may result in Gram-negative bacteria-related infections.

In our previous research, we determined ZG’s median lethal dose (LD_50_) and evaluated its safety on mice under different Sub-Lethal Doses (SLD) (Wang et al., 2024). Our findings (from the aforementioned previous experiments) provided the basis for selecting different ZG doses in this study. As a supplementary form of zinc, ZG plays a unique role in maintaining intestinal mucosal barrier integrity, regulating intestinal flora, and suppressing inflammatory responses (Lai et al., 2016). According to research, ZG can maintain intestinal mucosal barrier integrity and repair related injuries by promoting intestinal mucosal cell regeneration and regulating the expression of mucosal barrier-associated proteins (He et al., 2019). Additionally, ZG could exert antioxidant and anti-inflammatory effects (Lang et al., 2022). Furthermore, ZG could impact the richness and diversity of intestinal microorganisms, as well as the relative proportion of beneficial and harmful bacteria, via multiple channels, such as immune response regulation and production of antibacterial substances, thereby influencing the repair of intestinal mucosal barrier damage (Li et al., 2021; Samuelson et al., 2022). Studies have shown that zinc repairs intestinal mucosal damage by promoting intestinal tissue repair mechanisms, mitigating inflammation, and restructuring the gut microbiota to regulate mucosal integrity via modulating the NF-κB signaling pathway (Yang et al., 2020; Samuelson et al., 2022).

Previous studies on the repair of intestinal mucosal barrier damage were primarily based on a specific disease model and a single causative factor. In this regard, there are many unanswered questions regarding the mechanism underlying intestinal mucosal barrier damage, as well as the potential impact of treatment strategies such as the dual administration of antibiotics and LPS, which form the basis and motivation of this study. Herein, we simulated the actual disease situation by establishing a composite model of the dual induction of antibiotics and LPS-induced intestinal mucosal barrier damage in mice. Furthermore, we administered different ZG treatments to determine the optimal ZG dose and its efficacy in intestinal mucosal damage repair. In addition to elucidating the mechanism underlying intestinal barrier disorders, this study also provides new ideas and strategies for treating other related illnesses.

## 2 Materials and methods

### 2.1 Experimental animals

Healthy Specific Pathogen-Free (SPF)-grade male C57BL/6 J mice (aged = 6–8 weeks; average body weight = 20 ± 2 g) were sourced from the Experimental Animal Center of Guangxi Medical University (License number: SCXK Gui 2020-0003). All animal experimental procedures were approved by the Ethics Committee of Guangxi Medical University. One week before the experiment, the animals were acclimatized and housed in a barrier-level animal room with controlled temperature (22–25°C) and relative humidity (50–70%) and a 12 h/12 h light/dark cycle. During the experiment, the mice were fed an SPF-grade growth and reproduction diet and had *ad libitum* access to food and water.

### 2.2 Main reagents and drugs

Zinc gluconate (analytical grade, purity = 98%) was acquired from Macklin (Shanghai, China). Ampicillin (A9518-25G-9), neomycin sulfate (N6386-5G), metronidazole (M1547-25G), gentamicin sulfate (E003632-1 g), vancomycin (1404-93-9) and LPS (O55:B5, L2880-25MG) were all purchased from Sigma-Aldrich (United States, purity > 95%). The electron microscope fixative solution (G1102), Bovine Serum Albumin (BSA, GC305010), and the 4% paraformaldehyde fixative solution were purchased from Servicebio (Wuhan, China).

### 2.3 Main experimental instruments

The main experimental instruments included a high-speed low-temperature tissue grinder (Beijing, China), an ultra-trace Ultraviolet (UV) spectrophotometer (ThermoFisher Scientific, United States), an enzyme marker (Elx-808, Bio-Tek, United States), an electrophoresis and electrostatic transducer (Bio-Rad, USA), a Transmission Electron Microscope (TEM; HT7800, Hitachi), a dual-color infrared imaging system (ODYSSEY, Licor), an optical microscope (BX53F + DP73, OLYMPUS, Japan), and a 7,500 Real-Time Fluorescence Polymerase Chain Reaction (RT-PCR) Instrument (Applied Biosystems, United States).

### 2.4 Preparation of the antibiotic mixture

In our previous study, we successfully constructed an AAC mouse model via the Antibiotic cocktail (ABX) gavage approach (Yang et al., 2022). Herein, the ABX formulation scheme was as previously described (Hill et al., 2010; Liu J. et al., 2023). Specific ingredients included ampicillin (1 mg/mL), metronidazole (1 mg/mL), gentamicin (1 mg/mL), neomycin sulfate (1 mg/mL), and vancomycin hydrochloride (0.5 mg/ mL).

### 2.5 Preparation of the LPS solution

The LPS-induced intestinal infection was defined as in previous literature (Ding et al., 2004). The specific preparation approach was as follows. First, 25 mg LPS crystals were dissolved fully in 5 mL sterile physiological saline to obtain a stock solution with a concentration of 5 mg/mL. Subsequently, the solution was stored separately in a −20°C refrigerator in the dark. During the experiment, the appropriate amount of the stock solution was suctioned and diluted to obtain a final concentration of 0.5 mg/mL.

### 2.6 Preparation of the ZG solution

Herein, the ZG solution was prepared as previously described (Lai et al., 2016; Hsieh et al., 2017). Briefly, ZG was dissolved in ultrapure sterile water to obtain the Zn(C_6_H_11_0_7_)_2_ stock solution (with a concentration of 9.11 mg/mL). The stock solution was then sterilized using a 0.22 μm filter and stored in the dark at 4°C. To ensure mice from each dosage group received the same volume of the ZG solution, experimental concentrations were obtained with an equal-volume dilution of the stock solution. We previously determined the median lethal dose (LD_50_) of ZG and evaluated its safety in mice (Wang et al., 2024). Herein, 0.57 mg/mL, 1.14 mg/mL, and 3.42 mg/mL were selected as the low, medium, and high dose concentrations of the ZG solution, respectively.

### 2.7 Experimental grouping and the dosing regimen

We sourced 30 SPF-grade C57BL/6 J male mice and then acclimatized and fed them for 1 week before the experiment. The animals were randomly divided into five groups (*N* = 6): Normal Control (NC), model control (NS + ABX/LPS), low-dose ZG (ZG(L) + ABX/LPS), medium-dose ZG (ZG(M) + ABX/LPS), and high-dose ZG (ZG(H) + ABX/LPS). The NC group was left untreated throughout the experiment but received a normal diet and sufficient water. The remaining four groups were first subjected to continuous ABX gavage for 7 days (twice/day) to create an AAC mouse model. On day eight, LPS (5 mg/kg) was intraperitoneally injected to further induce the infection and exacerbate intestinal mucosal injury. Following that, on days 9, 11, and 13, the NS + ABX/LPS group received a tail vein injection of 0.9% Normal Saline (NS), while the ZG(L) + ABX/LPS, ZG(M) + ABX/LPS, and ZG(H) + ABX/LPS groups received tail vein injections of low (5.13 mg/kg), medium (10.26 mg/kg), and high (30.78 mg/kg) doses of the ZG solution, respectively.

**Figure 1:**
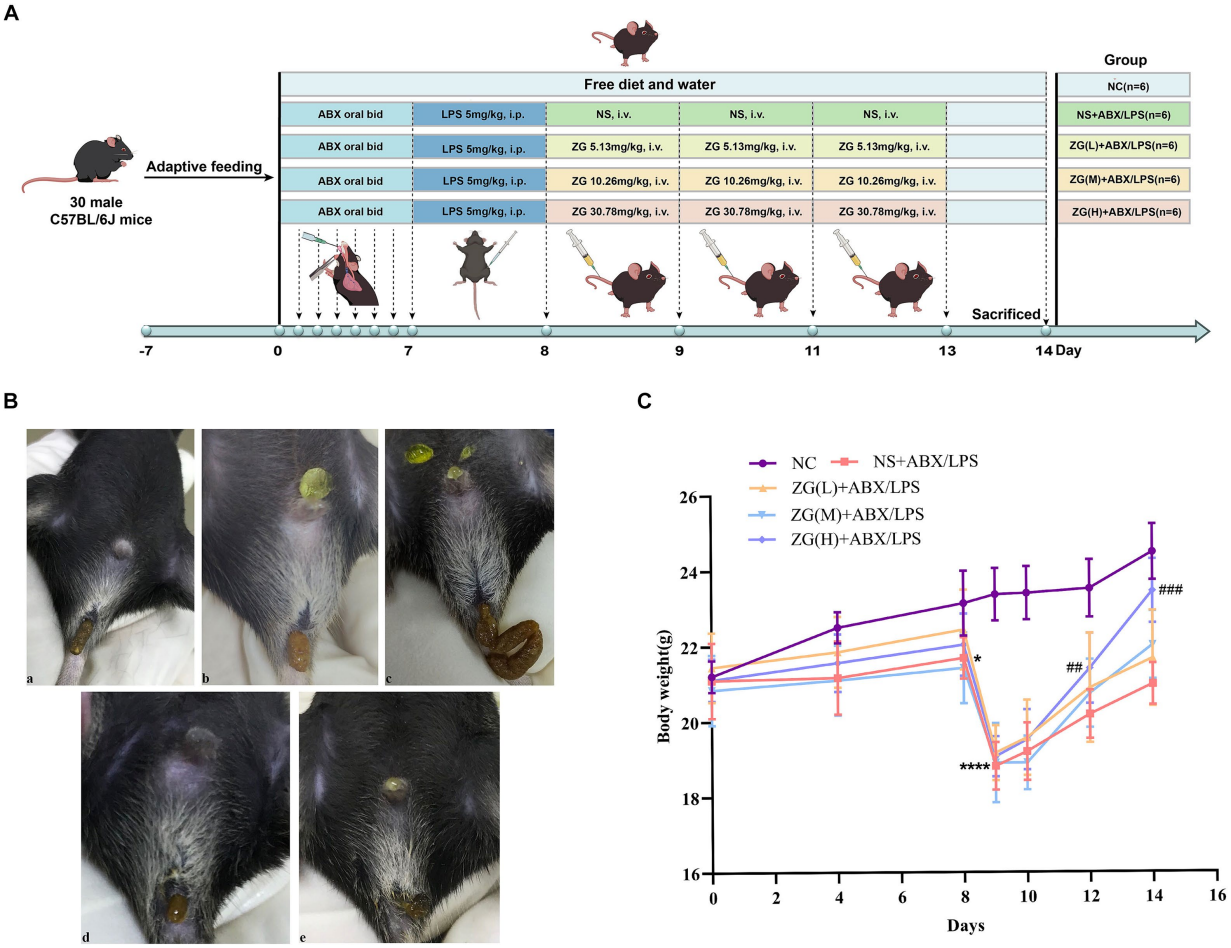
Experimental design protocol and changes in feces and body weight of mice during the experiment. **(A)** Animal experiment protocol and design. **(B)** Changes in feces of mice: (a) normal stool (b) Wet stools (c) Pasty stools (d) Semiliquid stools (e) Watery diarrhea. **(C)** Changes in body weight of mice. Compared with group NC: ^*^p < 0.05, ^****^p < 0.0001; Compared with group NS + ABX/LPS: ^##^*p* < 0.01, ^###^*p* < 0.001.

The injection doses of ZG and NS were both set at 9 mL/kg but were adjusted per body weight changes in experimental mice on each day as the study progressed. Mice in all groups received adequate food and water. The body weights of mice were determined and recorded on days 1, 4, 8, 9, 10, 12, and 14. Furthermore, diarrhea was assessed in mice at fixed time points each day after ABX gavage (days 4 and 8), after LPS administration (day 9), and after ZG treatments (days 10, 12, and 14). Figure 1A shows the study program and design.

### 2.8 Sample collection

On day 14, the animals were anesthetized via inhalation of 1.5% isoflurane. Following that, blood was collected by removing the eyeballs, separating the serum, and storing it at −80°C. Subsequently, the mice were euthanized via cervical dislocation. The intestinal segment 5 cm proximal to the ileocecal junction was dissected and cut into ~1–2 cm sample tissues. All intestinal contents were then gently washed with pre-cooled physiological saline, and a portion of the sample tissue was fixed in a 4% paraformaldehyde solution for Hematoxylin & Eosin (H&E) staining and Immunohistochemistry (IHC) analysis. Another portion was immersed in the electron microscope fixative solution for TEM observation. The remaining sample tissues were labeled and stored in portions at −80°C for Western Blotting (WB) and RT-PCR assays. Furthermore, the contents of the cecum were collected and immersed in liquid nitrogen for gut microbiological testing.

### 2.9 Ileum histopathology

Ileum specimens were obtained, routinely dehydrated, paraffin-embedded, sliced into 5 μm sections, deparaffinized with xylene, dehydrated with gradient alcohol, and subjected to H&E staining. The morphology of the intestinal tissues was then observed and photographed using a light microscope. The height of five intact villi (villus length, V) and the depth of five crypts (crypt depth, C) in each section were measured separately, and then the data were recorded. Following that, the chorionic ratio [Villous to Capillary Ratio (VCR or V/C)] was determined.

### 2.10 Transmission electron microscopy

Ileal specimens were obtained, sliced into 1 mm × 1 mm × 1 mm tissue blocks, rinsed and fixed in 0.1 M Phosphate Buffered Saline (PBS; PH7.4), dehydrated in gradient alcohol, osmotically embedded, polymerized, and ultra-thinly sliced (60–80 nm). Following that, the slices were stained with alcohol-saturated 2% uranyl acetate and 2.6% lead citrate solution and then visualized and photographed using TEM.

### 2.11 Immunohistochemistry

The paraffinized sections were recovered through heating to 98°C in 10 mM citrate buffer (PH 6. 0) for 10 min. Endogenous peroxidase was blocked with 10% (v/v) H_2_O_2_ for 30 min, while nonspecific antigens were blocked with serum at Room Temperature (RT) (20–25°C) for 30 min. The paraffinized sections were then incubated with rabbit anti-JAMA (1:400; GB111265-100; Servicebio, Wuhan, China) and rabbit anti-MLCK (1:400; GB113358-100; Servicebio, Wuhan, China) primary antibodies at 4°C overnight and then treated with Horseradish Peroxidase (HRP)-conjugated goat anti-rabbit IgG secondary antibodies (1:200; GB23303; Servicebio, Wuhan, China). Photographic images were captured and analyzed using an OLYMPUS microscope and Image-Pro Plus 6.0 software, respectively.

### 2.12 Real-time quantitative polymerase chain reaction

Total RNA was extracted from ~30 mg of colon tissue using NucleoZol reagent (740404.200, Gene Co. Ltd). Subsequently, RNA was quantified using a spectrophotometer at a wavelength of 260 nm. Complementary DNA (cDNA) was obtained via reverse transcription using the HiScript III RT SuperMix for qPCR (+gDNA wiper) Kit (R323-01, Vazyme, Nanjing, China). The ChamQ Universal SYBR qPCR Master Mix Kit (Q711-03, Vazyme, Nanjing, China) was used to perform qPCR on the ABI 7500 system (Applied Biosystems, CA, United States). Sangon Biotech Co. Ltd. (Shanghai) synthesized the primers used herein (Supplementary Table S1). The RT-QPCR data were analyzed using the 2^−ΔΔCT^ method, with the GAPDH gene expression as the endogenous control.

### 2.13 Western blotting

Total protein was isolated from the ileum and then quantified using the BCA Protein Assay kit. Protein (20 μg) from different samples was separated using 8 ∼ 12% Sodium Dodecyl Sulfate Polyacrylamide Gel Electrophoresis (SDS-PAGE) (Supplementary Table S2) and then transferred to Polyvinylidene Difluoride (PVDF) membranes. Supplementary Table S3 details the WB-related gene transfer conditions. The membranes were blocked with 5% skimmed milk for 4 h at RT and then probed overnight at 4°C with primary antibodies, including rabbit anti-ZO-1 (1:800, ab96587, Abcam), rabbit anti-Occludin [1:1,000, Cat #91131, Cell Signaling Technology (CST)], rabbit anti-Claudin-1 (1:1,500, AF0127, Affinity), rabbit anti-TLR4 (1:1,500, AF7017, Affinity), rabbit anti-NF-κB/p65 (1:2,000, Cat #8242, CST), and rabbit anti- β-actin (1:2,500, Cat #4970, CST), which was used as the loading control. Subsequently, the membranes were incubated with a goat anti-rabbit HRP-linked secondary antibody (1:10,000, Cat #bs-0295G-HRP, Bioss antibodies) for 1 h. The membranes were visualized using the Western BrightTM ECL kit (Advansta, CA, United States).

### 2.14 Enzyme-linked immunosorbent assay

The concentrations of DAO, D-LA, and ET in serum were determined using ELISA kits (FANKEW, Shanghai, China; and Shanghai Kexing Trading Co., Ltd., China) per the manufacturers’ instructions.

### 2.15 Intestinal flora 16S rRNA gene sequencing

Genomic DNA was extracted from the collected samples using the CTAB approach (Yu C. et al., 2023). A Nanodrop 2000 UV–Vis spectrophotometer (Thermofisher Scientific, Wilmington, MA, United States) and 1% agarose gel electrophoresis were used to examine the concentrations and quality of the DNA samples. The forward primer 341F (5′-CCTAYGGGRBGCASCAG-3′) and reverse primer 806R (5′-GGACTACNNGGGTATCTAAT-3′) were designed to amplify the V3 + V4 hypervariable regions of the 16S rDNA gene on a thermocycler PCR system. The PCR products were detected using 2% agarose gel electrophoresis and were gelled and recovered using an AxyPrep DNA gel recovery kit (Axygen Biosciences, Union City, CA, United States). Following quantification and homogenization, the DNA products underwent paired-end sequencing on an Illumina NovaSeq 6000 platform (Illumina, San Diego, CA, United States). Statistical analyses were performed using R (Version 2.15.3). Mice ileum contents were obtained via 16S rRNA high-throughput sequencing, commissioned by Wekemo Tech Group Co., Ltd. (Shenzhen, China).

### 2.16 Statistical analysis

Data were presented as Mean ± Standard Error of the Mean (mean ± SEM). The data were first collated using Excel 2019. One-way ANOVA was performed using SPSS27.0 software, and the Tukey method (for normally distributed data with equal variances) and the Kruskal-Wallis test (Dunn’s method; for non-normally distributed data) were used for multiple pairwise comparisons. The test value was set to *p* = 0.05, and results or differences with *p* < 0.05 were considered statistically significant. Drawings were generated using GraphPad Prism 9.0 software.

## 3 Results

### 3.1 Body weight changes in mice

Supplementary Table S4 and Figure 1C show the changes in body weight in mice during the experiment. No statistically significant difference (*F* = 0.29, *p* = 0.884) was found in baseline body weight (day 0) of mice in each group, indicating that they were comparable across all five groups. As the experiment progressed, the weight of mice in the NC group exhibited a continuous growth trend. In contrast, the overall trend of weight changes in the other four groups remained basically the same, although a decrease was noted on day 4, which later gradually increased to the baseline level by day 8. Furthermore, a linear decline was found on day 9, while a gradual increase was noted from day 10 onwards. Notably, the NC group exhibited a higher final body weight (day 14) compared with the other four groups.

On day 4, there was no significant change in body weight in the NS + ABX/LPS group compared with the NC group (*p* > 0.05). However, on day 8, the NS + ABX/LPS group exhibited a significant decrease in body weight compared with the NC group (*p* < 0.05), with a more significant reduction found on Day 9 (*p* < 0.0001). On day 10, no significant increase in the body weight of mice was noted in all the ZG treatment groups compared with the NS + ABX/LPS group (*p* > 0.05). Furthermore, on days 12 and 14, the ZG(H) + ABX/LPS group exhibited a significant increase in body weight compared with the NS + ABX/LPS group (*p* < 0.01 or *p* < 0.001). These results suggested that the concurrent induction of antibiotics and LPS-induced intestinal barrier damage had a substantial negative impact on the body weight of mice, representing an effect that was effectively reversed by ZG treatment.

### 3.2 Changes in mice feces

Figure 1B and Table 1 illustrate the changes in mice feces and diarrhea scores during the experiment, respectively. The NC group exhibited normal feces throughout the experiment. On the other hand, the NS + ABX/LPS group developed diarrhea from day 4 onwards, which manifested as a significant increase in diarrhea scores as the study progressed (*p* < 0.05). On the other hand, the ZG treatment group exhibited a significant reduction in diarrhea scores as the study progressed (*p* < 0.05).

**Table 1:** ZG reduced diarrhea scores in mice with the dual induction of antibiotics and LPS-induced diarrhea.

Day	NC	NS + ABX + LPS	*p*	ZG(L) + ABX/LPS	ZG(M) + ABX/LPS	ZG(H) + ABX/LPS	*p*
0	0 (0–0)	0 (0–0)	ns	–	–	–	–
4	0 (0–0)	2 (2–3)^*^	0.034	–	–	–	–
8	0 (0–0)	3 (2–3)^*^	0.034	–	–	–	–
9	0 (0–0)	3 (3–4)^*^	0.034	–	–	–	–
10	0 (0–0)	3 (3–4)	–	3 (3–4)	3 (3–4)	3 (3–4)	ns
12	0 (0–0)	2 (2–3)^**^	–	1 (1–2)^*^	1 (1–1)^#^	1 (1–1)^#^	0.016
14	0 (0–0)	2 (2–3)^**^	–	0 (0–1)^#^	0 (0–0)^##^	0 (0–0)^##^	0.026

Diarrhea scoring was based on changes in stool consistency and humidity: 0 = normal stool; 1 = wet stool; 2 = pasty stool; 3 = semiliquid stool; and 4 = watery diarrhea. Data were presented as median scores. The total score range was shown in parentheses. Data were analyzed using the Kruskal-Wallis test and Dunn’s *post-hoc* test. Compared with the NC group: ^*^p < 0.05,^**^p < 0.01; Compared with the NS + ABX/LPS group: ^#^*p* < 0.05, ^##^*p* < 0.01; ^ns^*p* > 0.05.

On day 10, no significant difference in diarrhea scores was identified between the ZG treatment group and the NS + ABX/LPS group. On day 12, diarrhea scores in the ZG(M) + ABX/LPS and ZG(H) + ABX/LPS groups significantly decreased (*p* < 0.05). On day 14, ZG treatment group exhibited significantly lower diarrhea scores (*p* < 0.05), with some demonstrating even greater reductions (*p* < 0.01). Compared with the NC group, diarrhea scores were significantly higher in the other four groups on day 10 (*p* < 0.05). On day 12, diarrhea scores were significantly elevated in the NS + ABX/LPS and ZG(L) + ABX/LPS groups (*p* < 0.05), and they were also significantly escalated in the NS + ABX/LPS group on day 14 (*p* < 0.05). Notably, the diarrhea scores in the ZG treatment groups were consistent with those in the NC group on day 14. These findings suggested that the dual induction of antibiotics and LPS-induced intestinal dysfunction caused diarrhea in mice, in which ZG treatment significantly ameliorated. However, the amelioration of diarrheal symptoms exhibited variability across different doses of ZG.

### 3.3 Pathological and morphological results of mouse ileum tissue

#### 3.3.1 The ileal mucosal integrity of mice in each group

**Figure 2:**
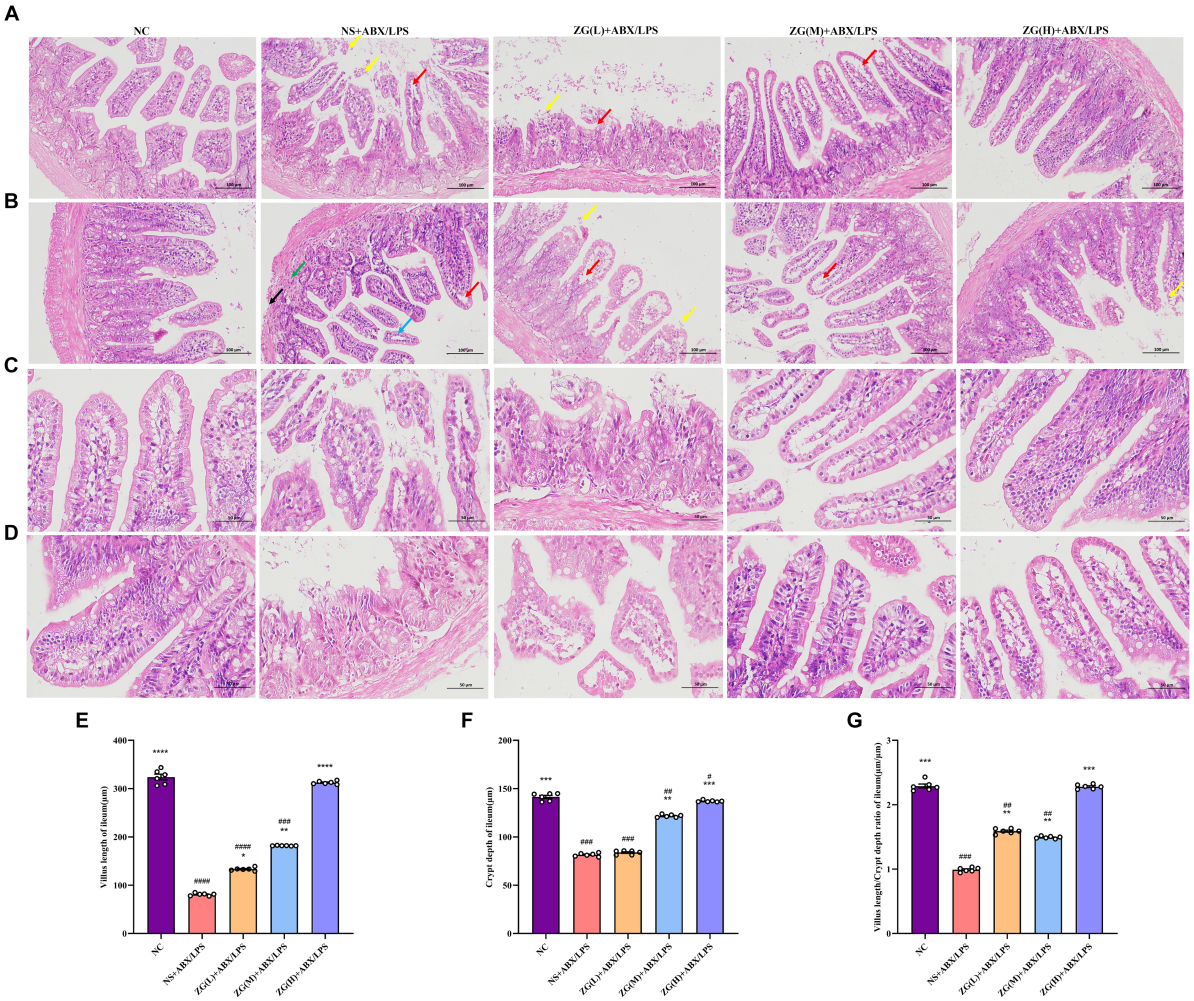
Representative images of histopathological and morphological changes in the ileum of each group of mice (HE staining, **A**,**B**: ×200, **C**,**D**: ×400). The detachment of intestinal villous epithelium is indicated by yellow arrows, while separation from the lamina propria and widening of the interstitial space are denoted by red arrows. Cytoplasmic laxity of intestinal villous epithelial cells is highlighted by blue arrows, granulocytic infiltration by green arrows, and disruption of the muscularis propria structure by black arrows. **(E)** Villus length **(F)** crypt depth **(G)** VCR. Compared with group NC: ^*^p < 0.05, ^**^p < 0.01, ^***^p < 0.001, ^****^p < 0.0001; Compared with group NS + ABX/LPS: ^#^*p* < 0.05, ^##^*p* < 0.01, ^###^*p* < 0.001, ^####^*p* < 0.0001.

The structural integrity of the mouse ileal mucosa following HE staining was assessed using a light microscope (Figures 2A–D). In the NC group, mice displayed a typical histological appearance of the ileum, characterized by an intact intestinal mucosa, normal glandular and crypt structures, and absence of epithelial cell shedding. Moreover, the intestinal villi exhibited a dense and well-arranged pattern, primarily composed of a single layer of columnar epithelium and goblet cells.

In contrast, NS + ABX/LPS mice showed severe damage to the ileal tissue mucosa, evidenced by significant shedding of intestinal villous epithelial cells (yellow arrow), separation between epithelium and lamina propria with widened gaps (red arrow), and partial loss of crypt structure. Additionally, the intestinal villus epithelial cells displayed a disorganized cytoplasm (blue arrow), accompanied by varying degrees of granulocyte infiltration (green arrow), disruption of the muscle layer structure (black arrow), and depletion of goblet cells. Comparatively, mice in the ZG (L) + ABX/LPS group exhibited a slight improvement in pathological ileum damage, accompanying by persistent separation of epithelium from lamina propria and widened gaps (red arrows). Moreover, partial detachment of intestinal villus epithelial cells (yellow arrows), loss of crypt architecture, and goblet cell depletion were observed. In the ZG(M) + ABX/LPS and ZG(H) + ABX/LPS groups, significant alleviation of pathological ileum damage was noted, which was characterized by the increased height of intestinal villi and depth of crypts. Occasional separation of epithelium from lamina propria (red arrow) and goblet cell proliferation were also evident.

#### 3.3.2 The villus height, crypt depth, and VCR of mice in each group

The height of the ileal villi, depth of crypts, and VCR values were further determined and analyzed in each group of mice under a light microscope (Figures 2E–G). Compared to the NC group, NS + ABX/LPS mice exhibited a significant decrease in ileal villus length, crypt depth, and VCR (*p* < 0.001), with certain animals showing even more remarkable reductions (*p* < 0.0001). Moreover, compared to the NS + ABX/LPS group, mice in the ZG treatment group exhibited significantly elevated ileal villus length, crypt depth, and VCR (*p* < 0.01), with some demonstrating even greater enhancements (*p* < 0.0001).

### 3.4 TEM observations of the mouse ileum

**Figure 3:**
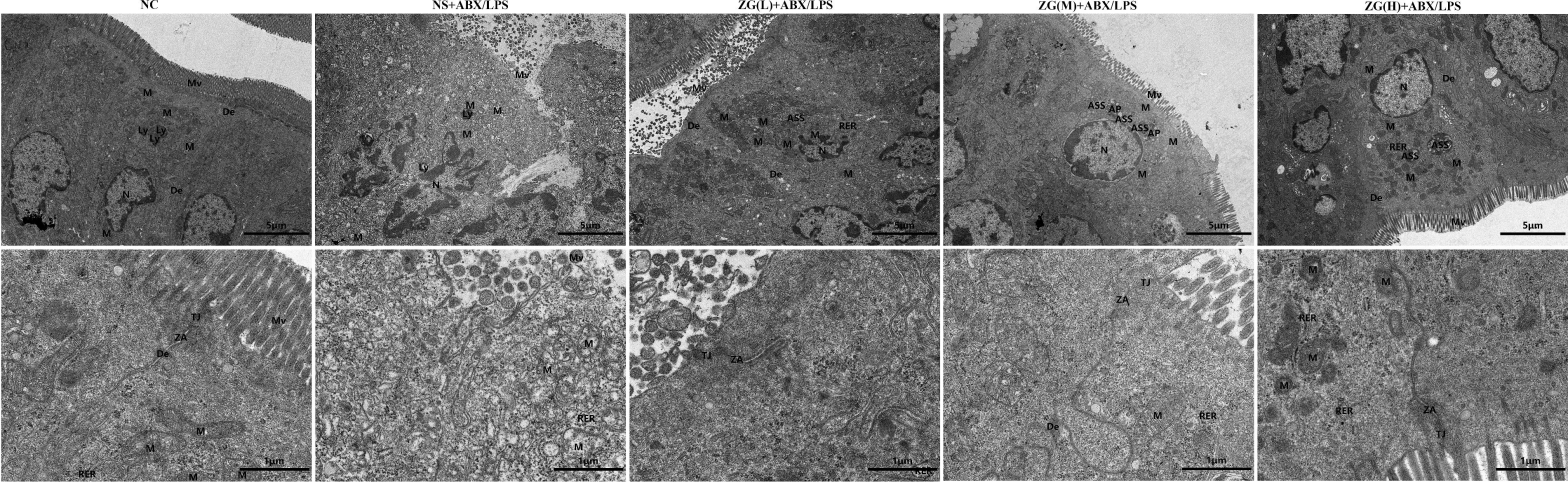
Representative images of ultrastructural changes of ileum in mice of each group (×8,000). Mv, Microvilli; TJ, Tight junction; ZA, Zonula Adherens; De, Desmosome; N, Nuclear; M, Mitochondria; Ly, Lysosome; ASS, Autolysosome; AP, Autophagosome; RER, Rough endoplasmic reticulum.

Ultrastructural changes in the mouse ileum tissue were observed under a TEM (Figure 3). Ileal IECs of NC mice were columnar, with a long, tightly arranged and neatly distributed microvilli morphology. Other notable features encompassed precisely delineated TJ and intermediate junction structures, as well as the presence of bridging granule formations. Furthermore, cellular gaps were tightly packed, with clearly defined epithelial cell membranes and nuclear membranes. The cell nuclei were regular, with homogeneous cytoplasmic distribution and scattered organelles. Additionally, mitochondria, rough endoplasmic reticulum (RER), and lysosomes were visible, with intact membrane structures. Conversely, the IECs of NS + ABX/LPS mice exhibited cone-shaped morphology, with numerous microvilli shedding and disappearing. Additionally, TJs, intermediate junctions, and desmosome structures were absent, accompanied by widened intercellular spaces, partial damage and dissolution of epithelial cell membranes, and decreased cytoplasmic density. Furthermore, the nucleus was sunken (with an abnormal shape), the mitochondria were swollen, the membrane structure was blurred, the matrix was partially dissolved and cavitated, and the cristae were broken and invisible. Additionally, the RER expanded, with ribosomes sparsely distributed on the endoplasmic reticulum (ER), and lysosomes were visible. Compared with the NS + ABX/LPS group, with the increase of the ZG treatment dose, the morphology of IECs was gradually improved, the microvilli were arranged neatly, the ileum structure was gradually restored, and the epithelial cell membrane gradually became intact. Furthermore, organelle structures, such as the nucleus, mitochondria, and RER were gradually improved. These findings demonstrated that ZG exerted a therapeutic effect on the dual induction of antibiotics and LPS-induced intestinal mucosal damage.

### 3.5 The effects of ZG on TJ-related gene and protein expression levels in IECs

#### 3.5.1 ZO-1 gene and protein expression levels

**Figure 4:**
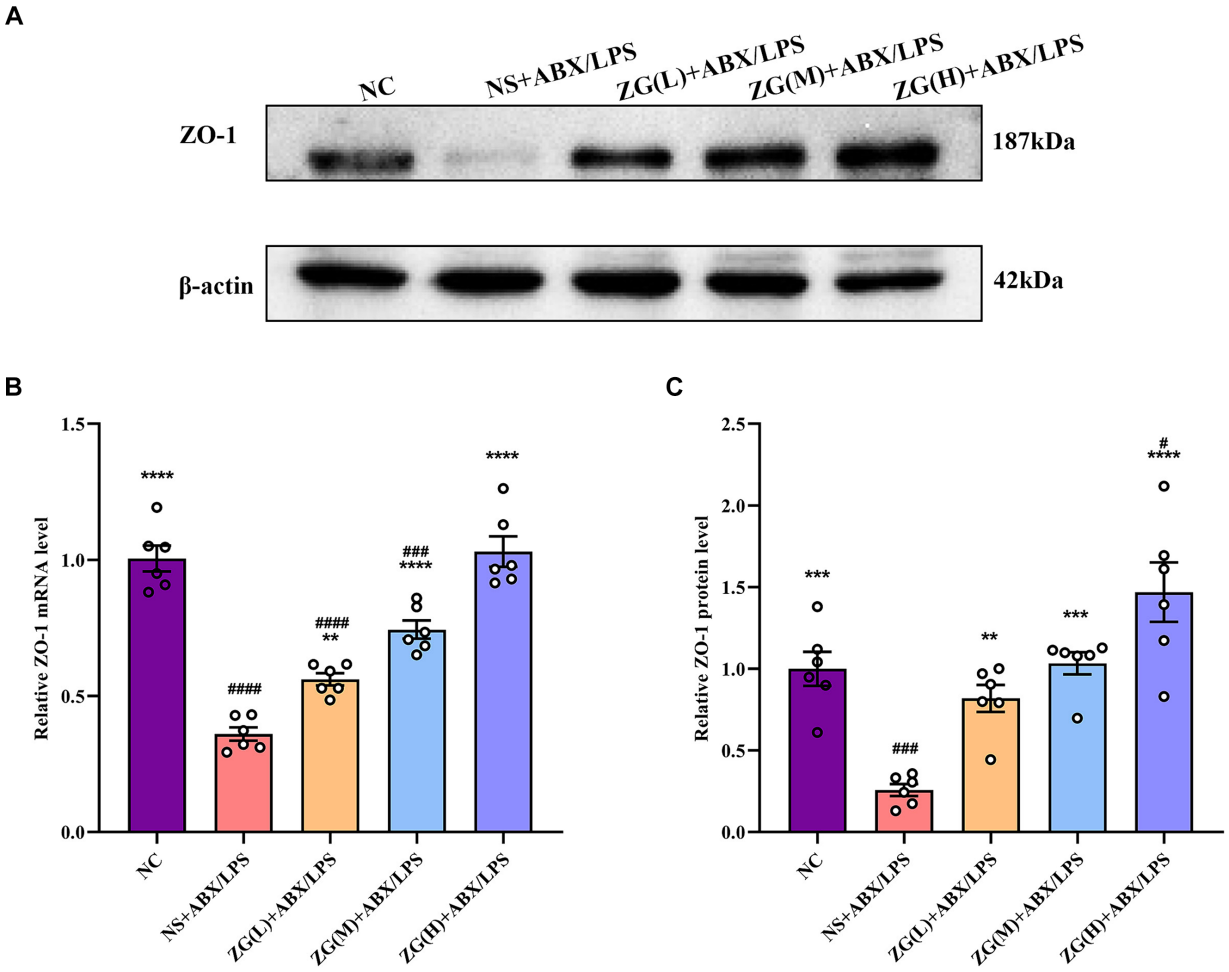
ZO-1 mRNA and protein relative expression levels in the ileum of mice in each group. Compared with group NC: ^**^p < 0.01, ^***^p < 0.001, ^****^p < 0.0001; Compared with group NS + ABX/LPS: ^#^*p* < 0.05, ^###^*p* < 0.001,^####^*p* < 0.0001. **(A-C)** The protein expression of tight junction protein (ZO-1) of ileum tissue in each group. **(B)** The mRNA expression of tight junction protein (ZO-1) of ileum tissues in each group.

Zonula Occludens-1 (ZO-1) is a TJ protein belonging to the MAGUK protein family. It is essential for maintaining and regulating intracellular TJ formation and function (Yu S. et al., 2023). According to the results of RT-PCR (Figure 4B), the ileal mucosa of NS + ABX/LPS mice exhibited a significantly lower ZO-1 mRNA expression level compared with that of NC mice (*p* < 0.0001). On the other hand, compared with the NS + ABX/LPS group, ZO-1 mRNA expression level was significantly higher in the ZG treatment group (*p* < 0.01), with some demonstrating even a higher expression level (*p* < 0.0001).

According to the results of WB (Figures 4A,C), the ZO-1 protein expression level was significantly lower in the NS + ABX/LPS group than that in the NC group (*p* < 0.001). Furthermore, compared with the NS + ABX/LPS group, the ZO-1 protein expression level was significantly higher in the ZG treatment group (*p* < 0.001), with some demonstrating even a higher expression level (*p* < 0.0001). Notably, ZG(H) + ABX/LPS exerted the greatest promotional effect on ZO-1 expression level. These findings suggested that the dual induction of antibiotics and LPS-induced mouse ileal mucosal injury significantly downregulated the ZO-1 gene expression level, impairing mucosal barrier function. On the other hand, different doses of the ZG treatment could significantly upregulate the ZO-1 gene expression level, especially at high doses (ZG(H) + ABX/LPS), indicating that ZG could exert a dose-dependent positive effect regarding the repair and enhancement of mucosal barrier function.

#### 3.5.2 Occludin gene and protein expression levels

**Figure 5:**
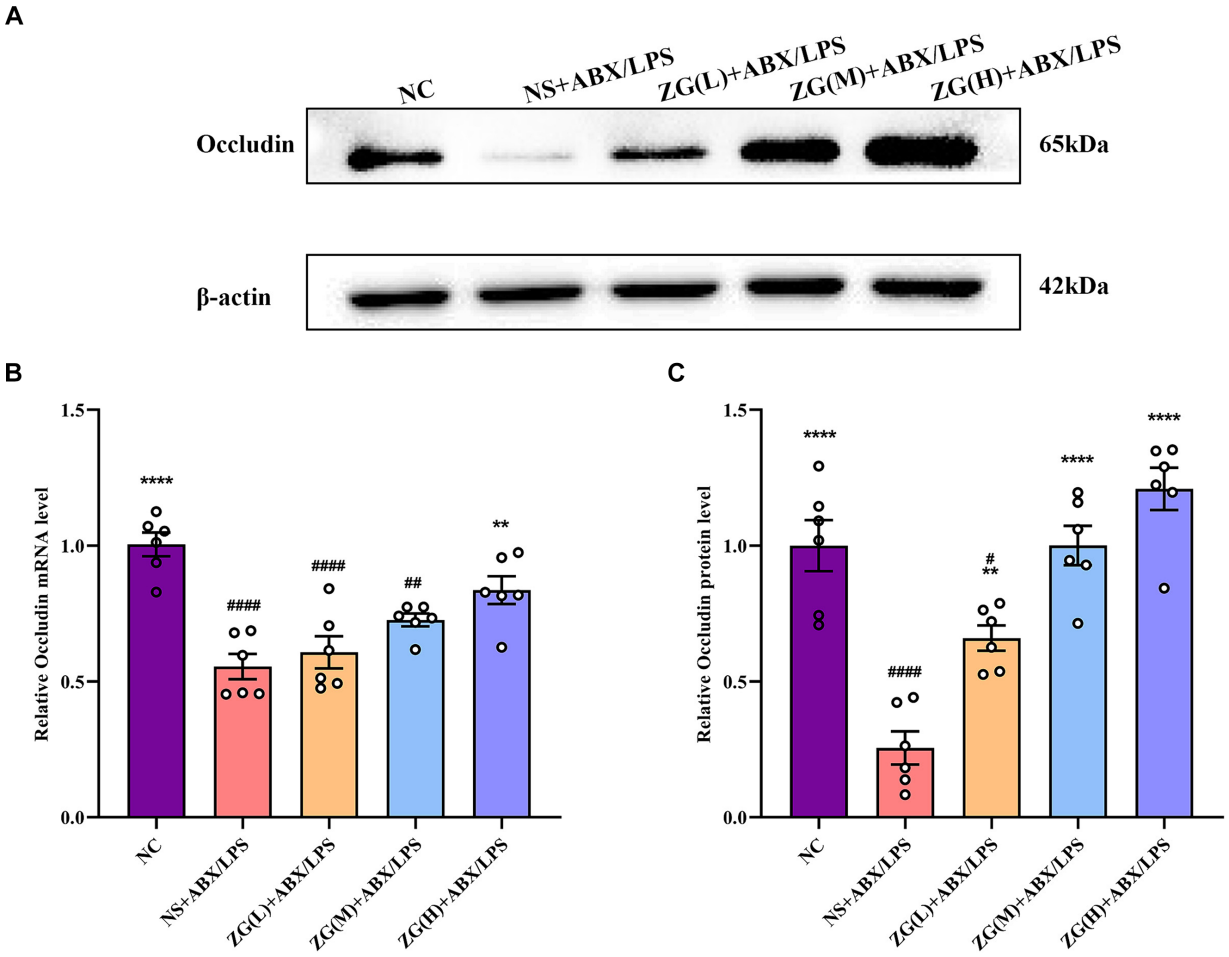
Relative expression levels of Occludin mRNA and protein in the ileum of mice in each experimental group. Compared with group NC: ^**^p < 0.01, ^****^p < 0.0001; Compared with group NS + ABX/LPS: ^#^*p* < 0.05, ^##^*p* < 0.01,^####^*p* < 0.0001. **(A-C)** The protein expression of tight junction protein (Occludin) of ileum tissue in each group. **(B)** The mRNA expression of tight junction protein (Occludin) of ileum tissues in each group.

Occludin, a crucial protein in intracellular connections, plays a critical role in their formation and stability. This gene facilitates the formation of TJs by interacting with other intracellular junction proteins, such as Claudin and ZO-1, thereby promoting the close alignment of IECs and minimizing gaps, ultimately enhancing intestinal barrier integrity (Suzuki, 2020; Kuo et al., 2022). According to the results of RT-PCR (Figure 5B), the ileal mucosal Occludin mRNA expression level was significantly lower in the NS + ABX/LPS group than that in the NC group (*p* < 0.0001). Furthermore, compared with the NS + ABX/LPS group, a significantly higher Occludin mRNA expression level was identified in the ZG(H) + ABX/LPS group (*p* < 0.01).

According to WB results (Figures 5A,C), Occludin protein expression level was significantly lower in the NS + ABX/LPS group than that in the NC group (*p* < 0.0001). Furthermore, compared with the NS + ABX/LPS group, the ZG treatment group exhibited a significantly higher Occludin protein expression level (*p* < 0.01), suggesting that ZG treatment has a beneficial effect on maintaining or restoring the integrity of the intestinal barrier, with some demonstrating even a higher expression level (*p* < 0.0001). Higher levels of Occludin indicate improved tight junction integrity, which can help prevent the passage of harmful substances from the gut lumen into the bloodstream, thereby protecting against intestinal barrier dysfunction and associated inflammatory responses. Notably, the ZG(M) + ABX/LPS and ZG(H) + ABX/LPS treatments exerted the greatest promotional effects on Occludin expression level, suggesting a dose-dependent response to ZG treatment. Thus, higher doses of ZG may lead to more pronounced improvements in intestinal barrier function, potentially due to increased bioavailability or enhanced pharmacological effects at higher concentrations. These findings indicate that the dual induction of antibiotics and LPS downregulated Occludin expression level in the ileal mucosa of mice, compromising the intestinal mucosal barrier. On the other hand, ZG treatment (especially the high-dose) significantly upregulated Occludin expression level in the ileal mucosa of mice, potentially contributing to the repair and preservation of the intestinal mucosal barrier.

#### 3.5.3 Claudin-1 gene and protein expression levels

**Figure 6:**
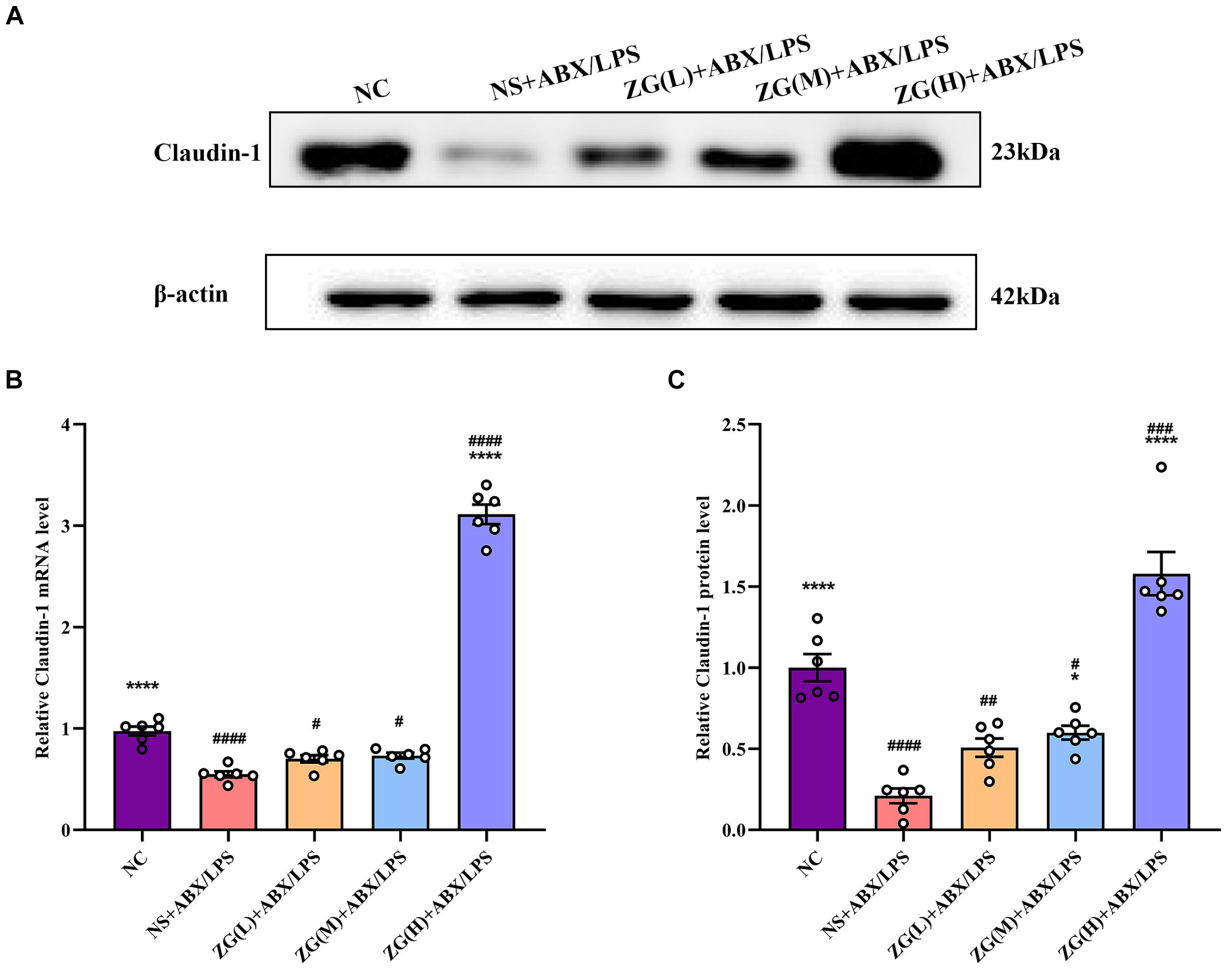
Claudin-1 mRNA and protein relative expression levels in the ileum of mice in each group. Compared with group NC: ^**^p < 0.01, ^***^p < 0.001, ^****^p < 0.0001; Compared with group NS + ABX/LPS: ^#^*p* < 0.05, ^##^*p* < 0.01, ^###^*p* < 0.001, ^####^*p* < 0.0001.

Claudin-1, an important member of the TJ protein family, is abundantly expressed in IECs and is crucially involved in maintaining intestinal barrier integrity (Masterson et al., 2019). According to the RT-PCR results (Figure 6B), ileal mucosal Claudin-1 mRNA expression level was significantly lower in the NS + ABX/LPS group than that in the NC group (*p* < 0.0001). Furthermore, compared with the NS + ABX/LPS group, the ZG(H) + ABX/LPS group had a significantly higher Claudin-1 mRNA expression level (*p* < 0.0001). Although the ZG(L) + ABX/LPS and ZG(M) + ABX/LPS groups exhibited a higher Claudin-1 mRNA expression level relative to the NS + ABX/LPS group, no statistically significant difference was noted (*p* > 0.05).

According to the WB results (Figures 6A,C), Claudin-1 protein expression level was significantly lower in the NS + ABX/LPS group than that in the NC group (*p* < 0.0001), suggesting impaired tight junction integrity in the intestinal mucosa of mice subjected to dual induction of antibiotics and LPS. Furthermore, compared with the NS + ABX/LPS group, the ZG treatment groups (except ZG(L) + ABX/LPS) exhibited a significantly higher Claudin-1 protein expression level (*p* < 0.05), with one group demonstrating even a higher expression level (*p* < 0.0001), highlighting that ZG has a protective effect on tight junction integrity, potentially by promoting the expression level of Claudin-1. Notably, ZG(H) + ABX/LPS group exerted the greatest promotional effect on Claudin-1 mRNA and protein expression levels. The upregulation of Claudin-1 expression by ZG may contribute to the preservation of tight junction structure and function, thereby preventing the leakage of harmful substances across the intestinal epithelium. These findings suggest that the dual induction of antibiotics and LPS severely damaged the structure and function of the intestinal mucosal barrier. However, after ZG treatment, especially with the high-dose (ZG(H) + ABX/LPS), Claudin-1 expression level was significantly upregulated, indicating that ZG promotes the restoration of intestinal barrier function.

#### 3.5.4 Junctional adhesion molecule A assay results

**Figure 7:**
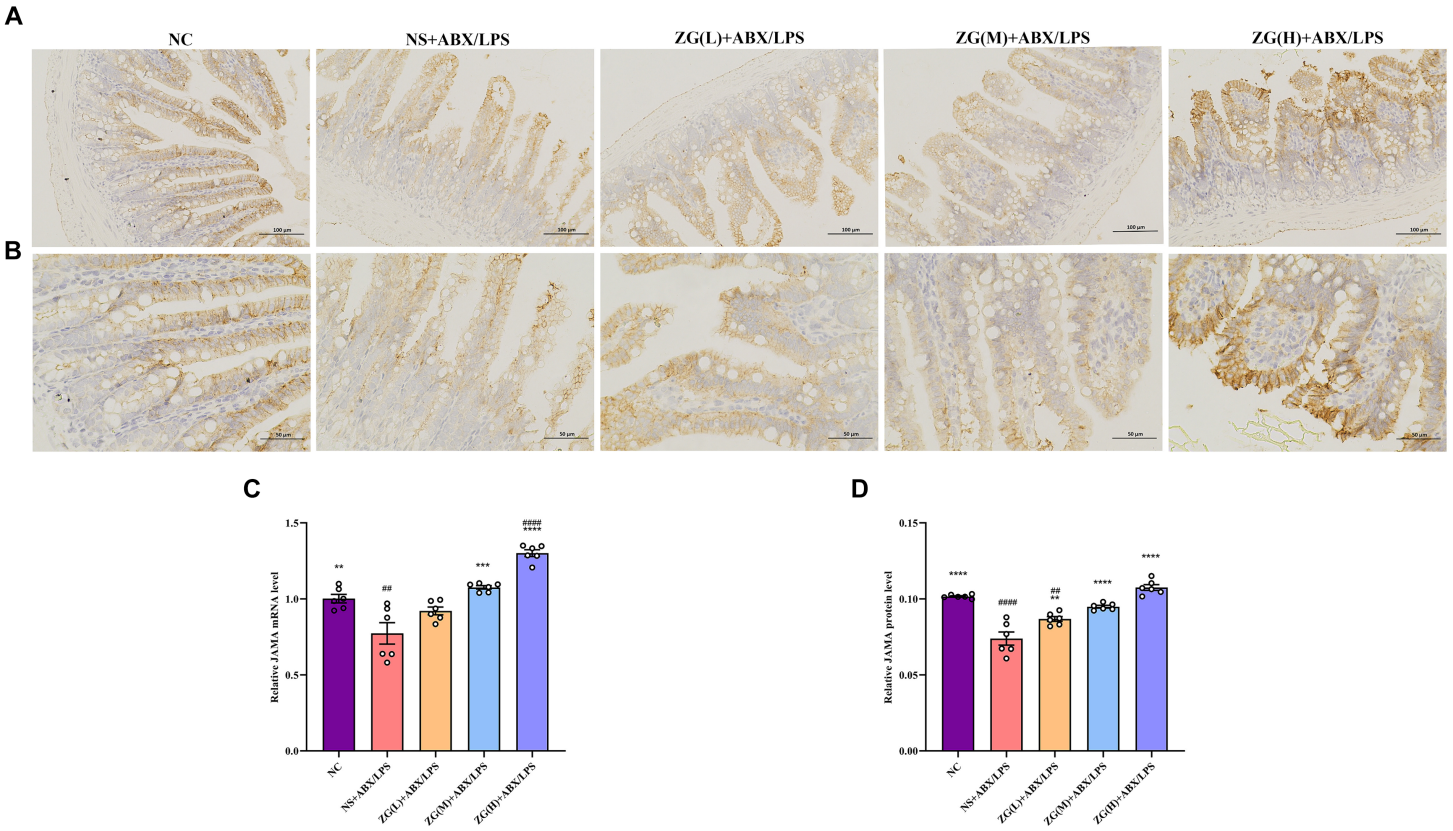
Representative images of JAM-A immunohistochemical staining of mouse ileum tissue (**A**: ×200, **B**: ×400). JAM-A mRNA **(C)** and protein **(D)** relative expression levels in the ileum of mice in each group. Compared with group NC:^**^p < 0.01, ^***^p < 0.001, ^****^p < 0.0001; Compared with group NS + ABX/LPS: ^##^*p* < 0.01, ^####^*p* < 0.0001.

JAM-A is a transmembrane glycoprotein belonging to the immunoglobulin superfamily that is predominantly found in the TJ of epithelial and endothelial cells. It is critically involved in the assembly and maintenance of the TJ and the establishment of epithelial cell polarity (Czubak-Prowizor et al., 2022). According to the RT-PCR results (Figure 7C), JAM-A mRNA expression level was significantly lower in the ileal mucosa of NS + ABX/LPS mice than that of NC mice (*p* < 0.01). Furthermore, compared with the NS + ABX/LPS group, ZG treatment groups [except ZG(L) + ABX/LPS] exhibited a significantly higher JAM-A mRNA expression level (*p* < 0.001), with one group demonstrating even a higher expression level (*p* < 0.0001).

Figures 7A,B illustrate that in NC mice, the JAM-A protein level was mainly expressed in the cytoplasm and cell membrane, and the positive cells were brown or brown granular and evenly distributed at the edge of IECs. This distribution pattern is indicative of intact tight junction structure and normal barrier function. Compared with NC mice, the staining of JAM-A in NS + ABX/LPS mice exhibited non-uniform distribution or fading. This alteration in staining pattern suggests disruption or downregulation of JAM-A protein expression, which is consistent with impaired TJ integrity and compromised intestinal barrier function. According to the IHC results (Figure 7D), JAM-A protein expression level was significantly lower in the NS + ABX/LPS group than that in the NC group (*p* < 0.0001). This downregulation of JAM-A expression unveiled the detrimental effect of dual induction of antibiotics and LPS on TJ proteins and intestinal barrier integrity. Furthermore, compared with the NS + ABX/LPS group, JAM-A protein expression level was significantly higher in ZG treatment groups (*p* < 0.001), with some demonstrating even a higher expression level (*p* < 0.0001). This restoration of JAM-A expression suggests that ZG has a protective effect on tight junction integrity, potentially by promoting the expression of JAM-A. These findings suggest that ZG may exert a protective effect against the dual induction of antibiotics and LPS-induced ileal mucosal barrier damage in mice and help maintain or increase JAM-A expression level, promoting the stability and repair of the intestinal mucosal barrier.

#### 3.5.5 Myosin light chain kinase assay results

**Figure 8:**
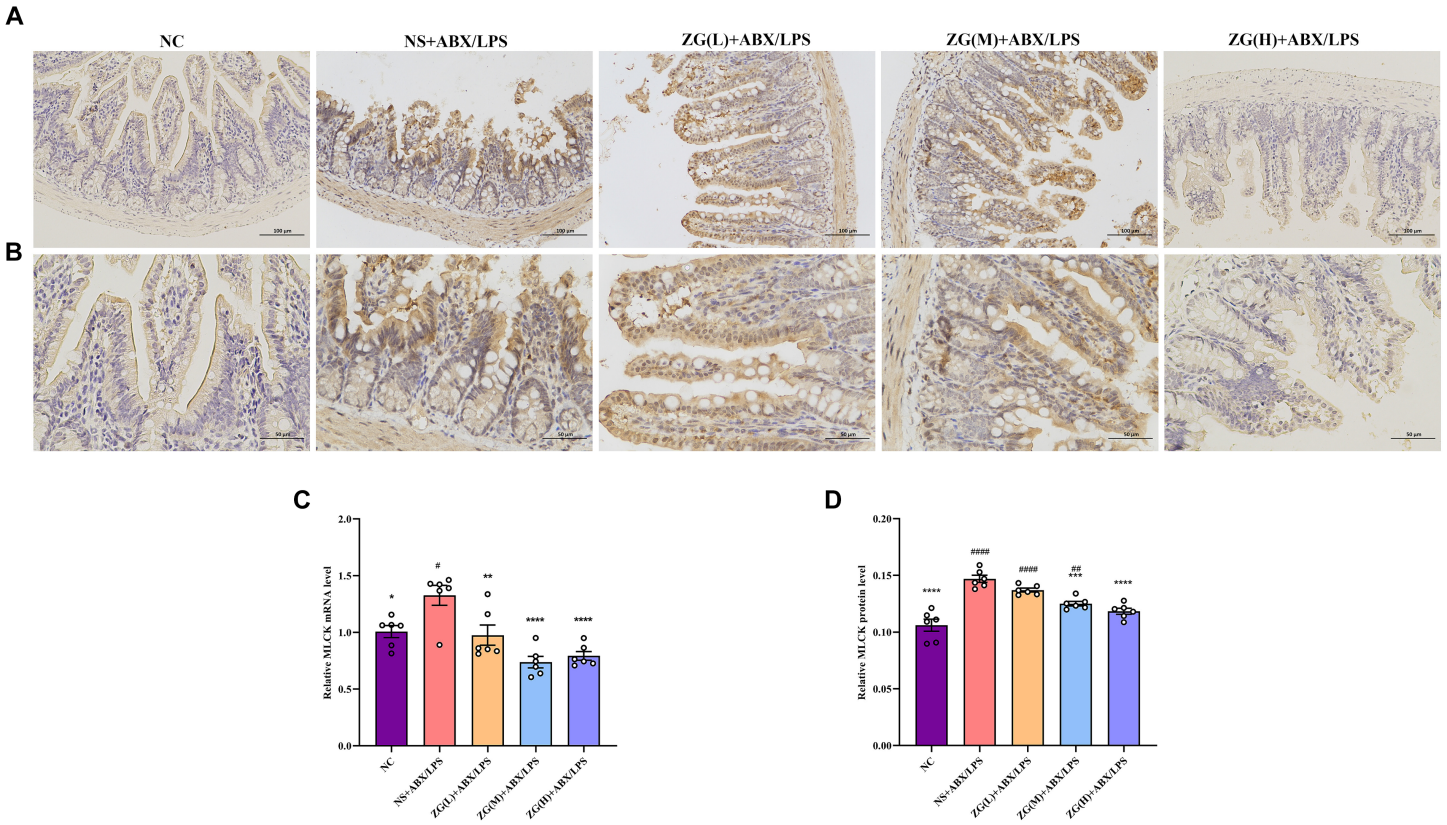
Representative images of MLCK immunohistochemical staining of mouse ileum tissue (**A**: ×200, **B**: ×400). MLCK mRNA **(C)** and protein **(D)** relative expression levels in the ileum of mice in each group. Compared with group NC: ^*^p < 0.05, ^**^p < 0.01, ^***^p < 0.001, ^****^p < 0.0001; Compared with group NS + ABX/LPS: ^#^*p* < 0.05, ^##^*p* < 0.01, ^####^*p* < 0.0001.

MLCK is a protein kinase that regulates actin contraction and relaxation in IECs via actin phosphorylation, affecting cell shape, intercellular junction stability, and membrane permeability. Furthermore, MLCK regulates inflammatory processes (Huang et al., 2020; Chihade et al., 2023). According to the RT-PCR results (Figure 8C), compared with that of NC mice, MLCK mRNA expression level in the ileal mucosa of NS + ABX/LPS mice was significantly higher (*p* < 0.05). Furthermore, compared with the NS + ABX/LPS group, MLCK mRNA expression level was significantly lower in the ZG treatment groups (*p* < 0.01), with some groups demonstrating even a lower expression level (*p* < 0.0001).

Figures 8A,B illustrate that in NC mice, MLCK protein expression level was mainly in the cytoplasm and cell membrane, and the positive cells were brownish-yellow or brownish granular and uniformly distributed at the edges of IECs. Compared with NC mice, the MLCK staining in NS + ABX/LPS mice displayed irregular distribution or attenuation. According to the IHC results (Figure 8D), MLCK protein expression level was significantly higher in the NS + ABX/LPS group than that in the NC group (*p* < 0.0001). Furthermore, compared with the NS + ABX/LPS group, MLCK protein expression level was significantly lower in the ZG treatment groups (except ZG(L) + ABX/LPS) (*p* < 0.001), with one group demonstrating even a lower expression level (*p* < 0.0001). These findings suggest that the dual induction of antibiotics and LPS-induced intestinal damage could be associated with MLCK overexpression, and ZG could ameliorate the dual induction of antibiotics and LPS-induced intestinal mucosal injury by regulating MLCK expression level.

### 3.6 Differences in ZG impact on TLR4 and NF-κB/p65 protein expression levels in mice

The TLR4/NF-κB signaling pathway is an essential immune response regulatory system that is involved primarily in the perception and response to external pathogens (e.g., bacterial LPS, viral RNA, etc.) *in vivo*. In the intestines, this signaling pathway plays a critical role in maintaining intestinal barrier integrity and immune homeostasis (Samuelson et al., 2022).

**Figure 9:**
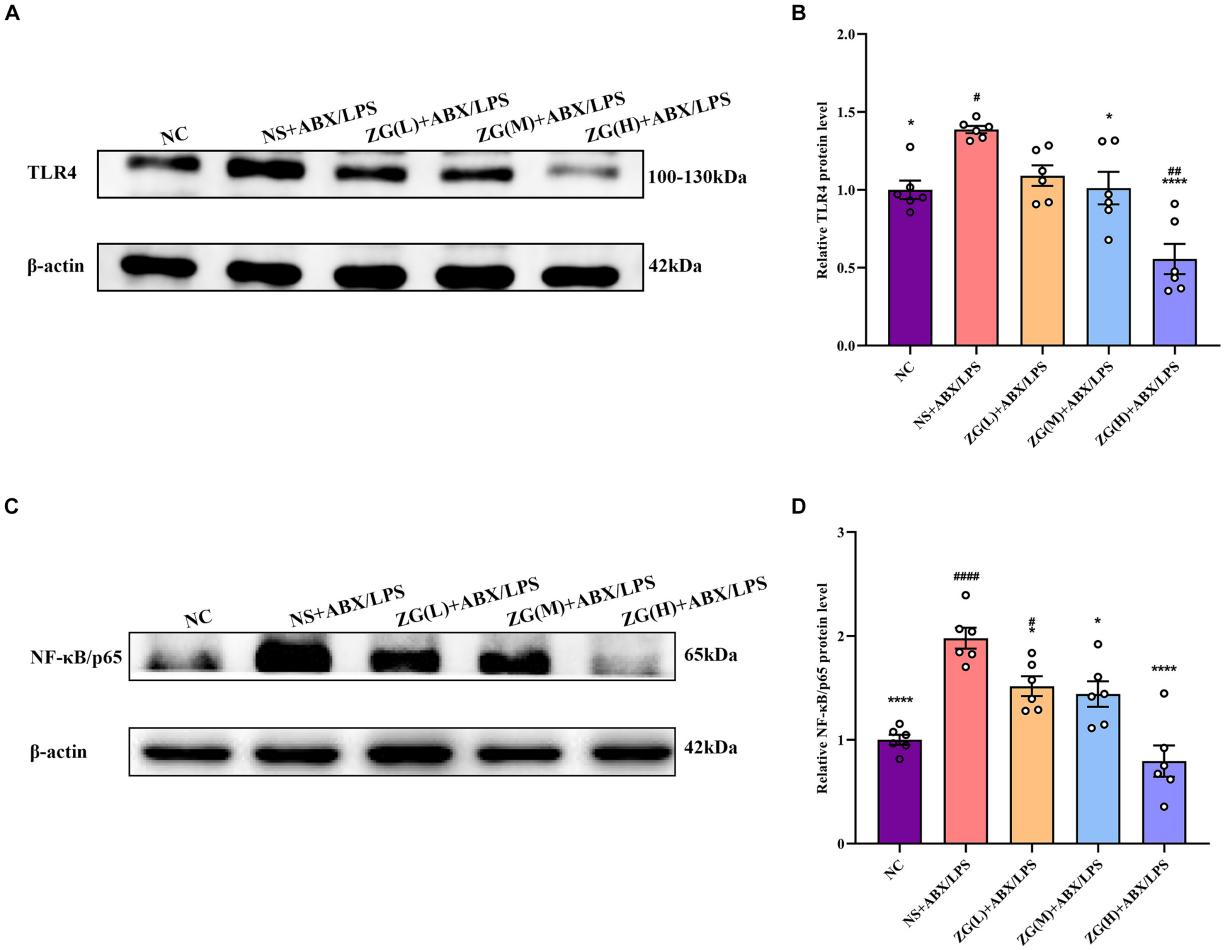
Comparison of variations in the relative expression levels of TLR4 and NF-κB protein in ileum tissues of mice in various groups. Compared with group NC: ^*^p < 0.05, ^****^p < 0.0001; Compared with group NS + ABX/LPS: ^#^*p* < 0.05,^##^*p* < 0.01,^####^*p* < 0.0001. **(A-B)** The protein expression of TLR4/NF-κB signaling pathway protein (TLR4) of ileum tissue in each group. **(C-D)** The protein expression of TLR4/NF-κB signaling pathway protein (NF-κB/p65) of ileum tissue in each group.

According to TLR4 WB results (Figures 9A,B), TLR4 protein expression level was significantly higher in the NS + ABX/LPS group than that in the NC group (*p* < 0.05). Furthermore, compared with the NS + ABX/LPS group, TLR4 protein expression level was significantly lower (*p* < 0.05) in the ZG treatment groups (except ZG(L) + ABX/LPS), with one group demonstrating even a lower expression level (*p* < 0.0001). Notably, ZG(H) + ABX/LPS exerted the greatest inhibitory effect on TLR4 protein expression level. According to NF-κB/p65 WB results (Figures 9C,D), NF-κB/p65 protein expression level was significantly higher in the NS + ABX/LPS group than that in the NC group (*p* < 0.0001). Furthermore, compared with the NS + ABX/LPS group, the ZG treatment groups exhibited a significantly lower NF-κB/p65 protein expression level (*p* < 0.05), with some demonstrating even a lower expression level (*p* < 0.0001). Notably, ZG(H) + ABX/LPS exerted the greatest inhibitory effect on NF-κB/p65 protein expression level. These findings suggest that the dual induction of antibiotics and LPS-induced ileal mucosal injury in mice could be associated with the activation of TLR4 and NF-κB/p65 protein levels, and ZG could play an anti-inflammatory and barrier-protective role against antibiotics and LPS-induced intestinal mucosal injury.

### 3.7 Mouse serum levels of DAO, D-LA, and ET

Measurement of intestinal mucosal permeability can reveal the integrity and maturity of the gut. Increased intestinal mucosal barrier permeability may reflect disrupted barrier function. To assess this impairment, the levels of DAO, D-LA, and ET in mouse serum were quantified by ELISA.

**Figure 10:**
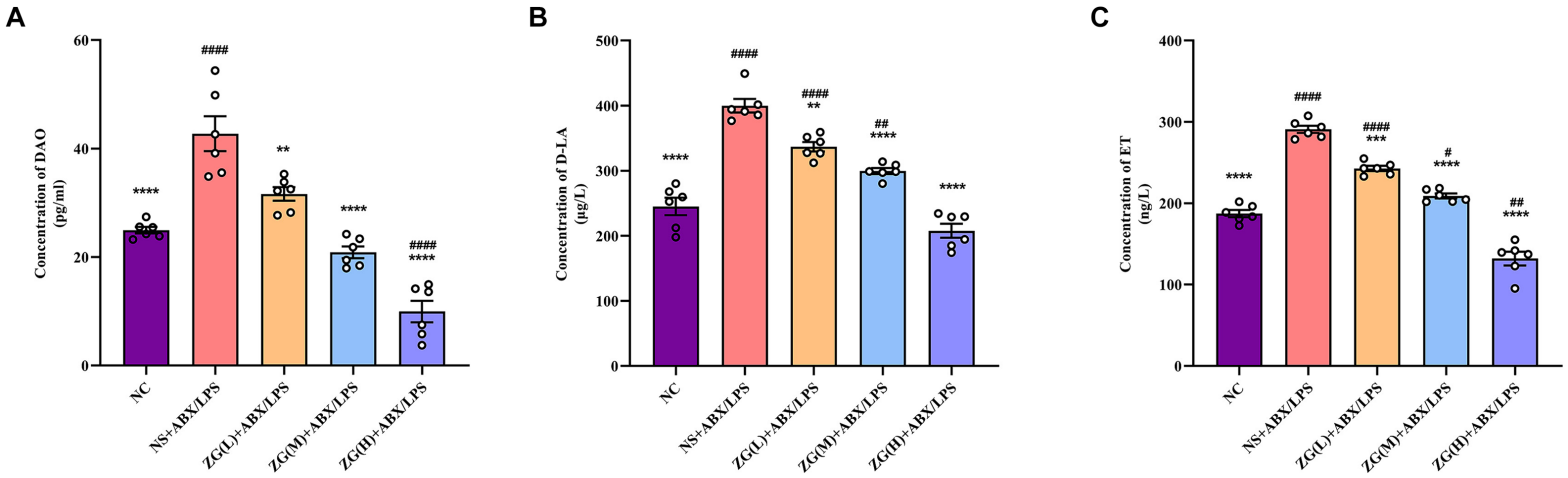
Changes of DAO, D-LA and ET levels in serum of mice in each group. Compared with group NC: ^**^p < 0.01, ^***^p < 0.001, ^****^p < 0.0001; Compared with group NS + ABX/LPS: ^#^*p* < 0.05,^##^*p* < 0.01,^####^*p* < 0.0001. **(A-C)** Concentration of DAO, D-LA and ET in mouse serum. They are the main biomarkers of intestinal mucosal permeability and indirectly reflect intestinal mucosal barrier damage.

Figure 10A indicates that compared with NC mice, the DAO content in the serum of NS + ABX/LPS mice was extremely significantly elevated (*p* < 0.0001). Compared with NS + ABX/LPS group, the DAO content in the serum of mice in each ZG treatment group was significantly or extremely significantly lower (*p* < 0.01 or *p* < 0.0001), reaching the lowest in the ZG(H) + ABX/LPS group. As illustrated in Figure 10B D-LA content in the serum of NS + ABX/LPS mice was extremely significantly higher than that in NC mice (*p* < 0.0001). Moreover, D-LA content in the serum of mice in each ZG treatment group was significantly or extremely significantly lower compared with the NS + ABX/LPS group (*p* < 0.01 or *p* < 0.0001), reaching the lowest in the ZG(H) + ABX/LPS group. Figure 10C showed that compared with NC, the ET content in the serum of NS + ABX/LPS mice was extremely significantly higher (*p* < 0.0001). The ET content in the serum of mice in each ZG treatment group was extremely significantly lower compared with the NS + ABX/LPS (*p* < 0.001 or *p* < 0.0001), being lowest in the ZG(H) + ABX/LPS. These findings indicate that the combined induction of antibiotics and LPS disrupted the intestinal barrier, resulting in heightened permeability of the intestinal mucosal barrier and inflammation. Treatment with ZG may alleviate the rise in intestinal mucosal barrier permeability and alleviate intestinal inflammation.

### 3.8 Results of high-throughput sequencing analysis of 16S rRNA in mouse intestinal flora

#### 3.8.1 Species diversity dilution curve

**Figure 11:**
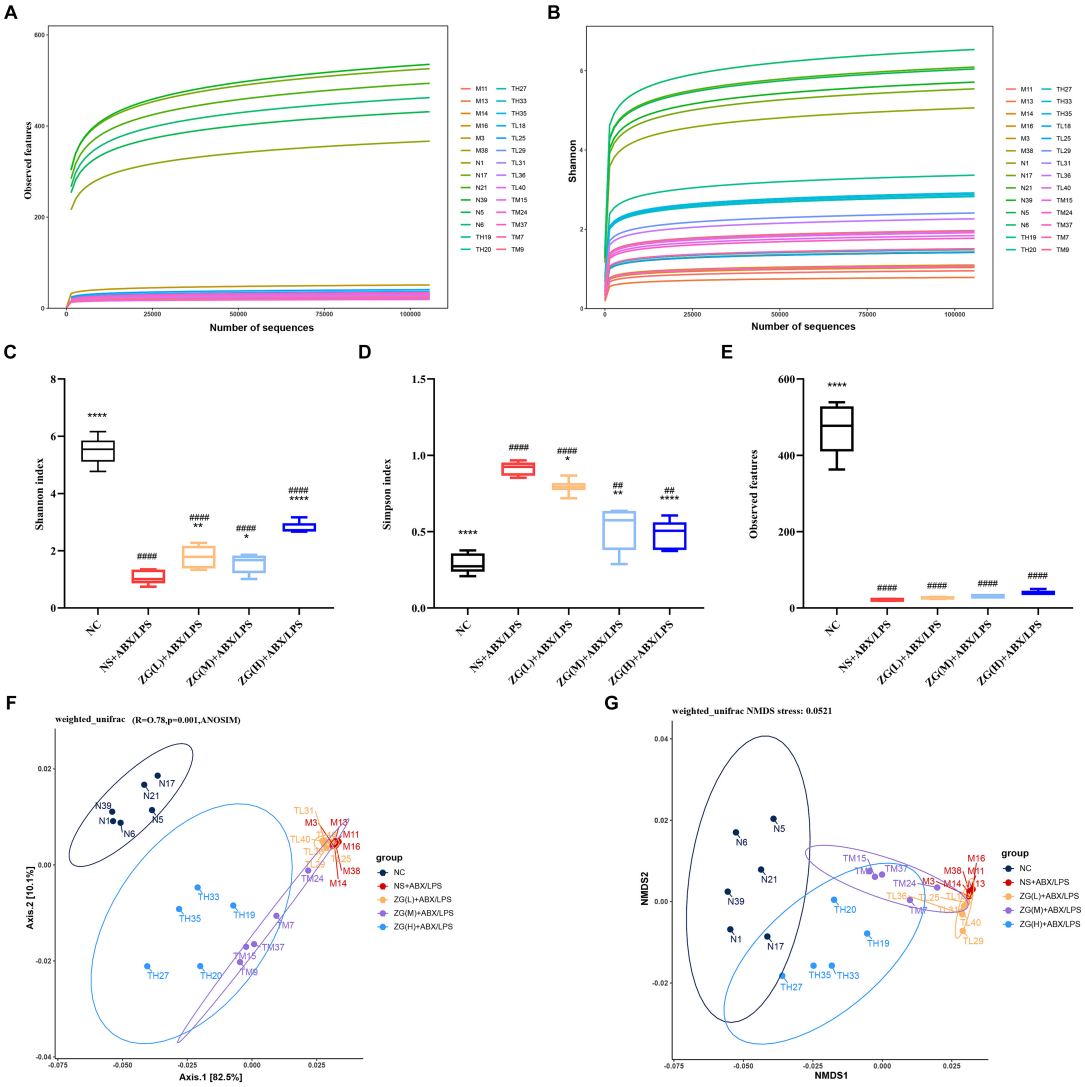
Diversity and composition of gut microbiota. **(A,B)** Species diversity rarefaction curve. **(C–E)** Alpha diversity was evaluated by the Shannon, Simpson, and Observed features index. Weighted UniFrac analysis was utilized to distinguish bacterial clustering. **(F)** PCoA, **(G)** NMDS. Compared with group NC: ^*^p < 0.05, ^**^p < 0.01, ^****^p < 0.0001; Compared with group NS + ABX/LPS: ^##^*p* < 0.01, ^####^*p* < 0.0001.

Observed features dilution curve is used to assess whether new species can still be observed when the number of sequences increase. Figure 11A illustrates that as the number of sequence sequencing increases, the curve tends to be saturated, suggesting that new samples no longer introduce new species. The Shannon diversity dilution curve considers the relative abundance of species and more comprehensively reflects the diversity of bacterial community results. The curve in Figure 11B demonstrates that diversity tends to stabilize as the number of sequence sequencing increases. The dilution curve analysis of species diversity reveals that the curve for each sample remains relatively flat, indicating that the sequencing depth adequately covers all species present in each sample. This suggests that the bacterial community structure is accurately reflected by the sequencing data. The experimental data can be used for further analysis.

#### 3.8.2 Alpha diversity index analysis

Alpha diversity is often employed to analyze the diversity and richness of microbial communities within a sample (within-community). Indices such as Shannon, Simpson, and Observed features are evaluated to assess the species diversity of a given sample (Xu et al., 2023). The Shannon index integrates species richness and relative abundance, offering a holistic assessment of overall species composition. A higher Shannon index signifies increased species richness and more balanced relative abundance. Data presented in Figure 11C show that compared with the NC group, the Shannon index of the NS + ABX/LPS group was extremely significantly lower (*p* < 0.0001), suggesting that the diversity of the bacterial flora in the NS + ABX/LPS group was decreased. The Shannon index of each ZG treatment group was significantly or extremely significantly higher compared with the NS + ABX/LPS group (*p* < 0.05 or *p* < 0.01 or *p* < 0.0001), suggesting that the ZG treatment increased the diversity of bacterial flora.

Reduced values of Simpson’s index indicate greater species richness and evenness within the sample. Conversely, a higher Simpson’s index may indicate a microbiota where a few dominant species predominate, whereas a lower index suggests a more evenly distributed array of species. Figure 11D demonstrates that the Simpson’s index was significantly or highly significantly lower in the NC group and each treatment group of ZG relative to that of the NS + ABX/LPS group (*p* < 0.05 or *p* < 0.01 or *p* < 0.0001).

The Observed features index refers to the number of ASVs which are measured in a specific sample and reflects the abundance of ASVs in the sample. Figure 11E shows that the Observed features index was extremely significantly lower in the NS + ABX/LPS group than in the NC group (*p* < 0.0001), indicating that the ASV abundance of the flora in the NS + ABX/LPS group was much lower relative to that in the NC group. Compared with the NS + ABX/LPS group, the Observed features index showed an increasing trend in each treatment group of ZG, but not significantly (*p* > 0.05).

#### 3.8.3 Beta diversity comparative analysis

Beta Diversity is frequently utilized to compare microbial community structures among samples. In this study, we employed the Weighted Unifrac algorithm distance and utilized R software to conduct Principal Component Analysis (PCoA) and Non-Metric Multi-Dimensional Scaling (NMDS) analyses (Figures 11F,G). Two tests were run to analyze the gut bacteria in the mice. Interestingly, both tests showed similar results. The bacteria in the guts of the mice in each group (regular, healthy mice, mice with the induced condition, and mice treated with the drug) were all different and distinct from each other. This suggests that both the condition itself and the drug treatment significantly impacted the composition of the gut bacteria. The ANOSIM analysis value was *R* = 0.78, *p* = 0.001, which further demonstrated that there were significant differences in the composition of cecal microorganisms in mice among different groups (*p* < 0.05).

**Figure 12:**
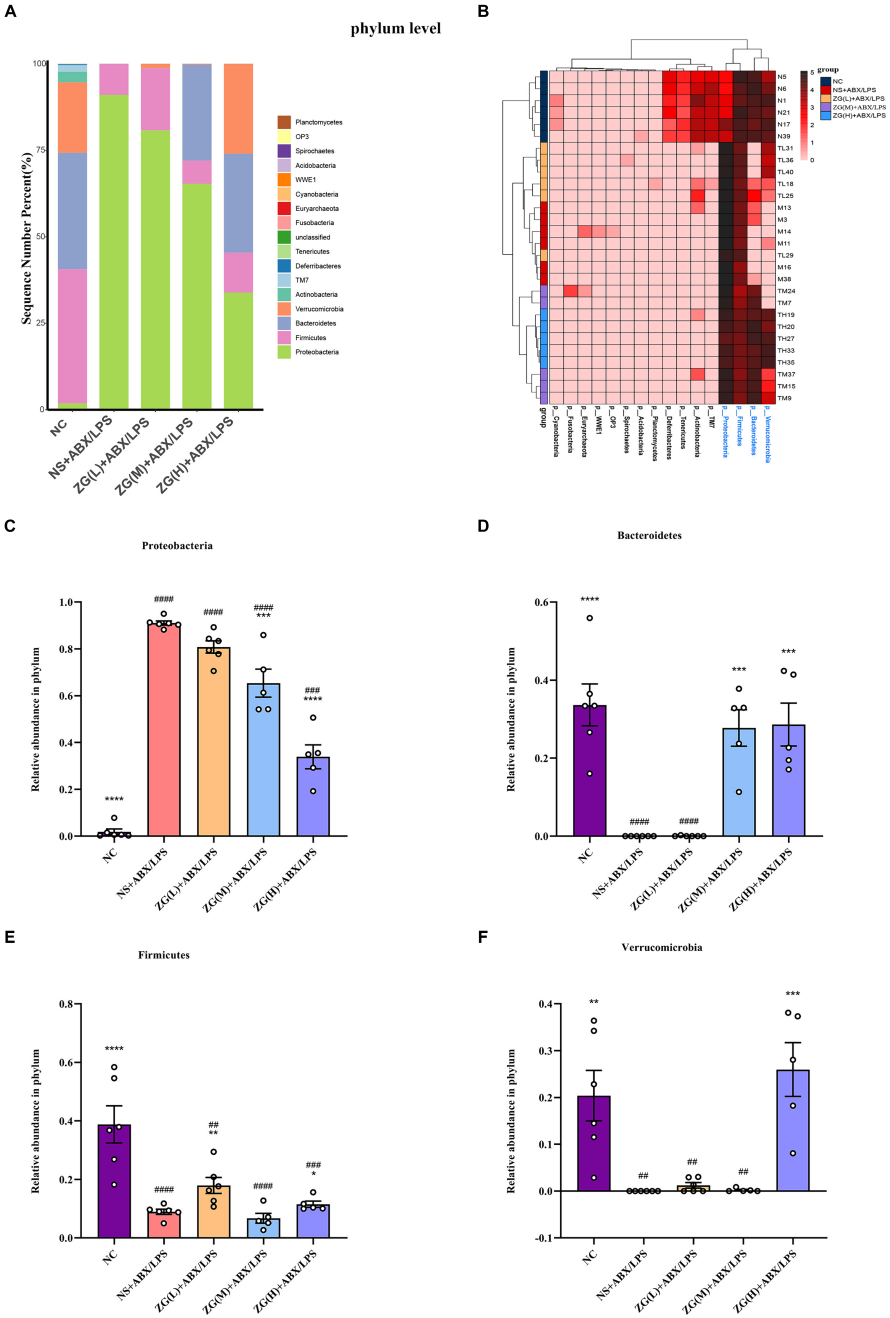
The distribution of cecal content microflora in each group of mice categorized at the phylum level. **(A)** Histogram of relative abundance ratios. **(B)** Heat map of absolute abundance clustering. **(C–F)** Histogram of relative abundance of the dominant bacterial phylum. Compared with group NC: ^*^p < 0.05, ^**^p < 0.01, ^***^p < 0.001, ^****^p < 0.0001; Compared with group NS + ABX/LPS: ^##^*p* < 0.01, ^###^*p* < 0.001,^####^*p* < 0.0001.

**Figure 13:**
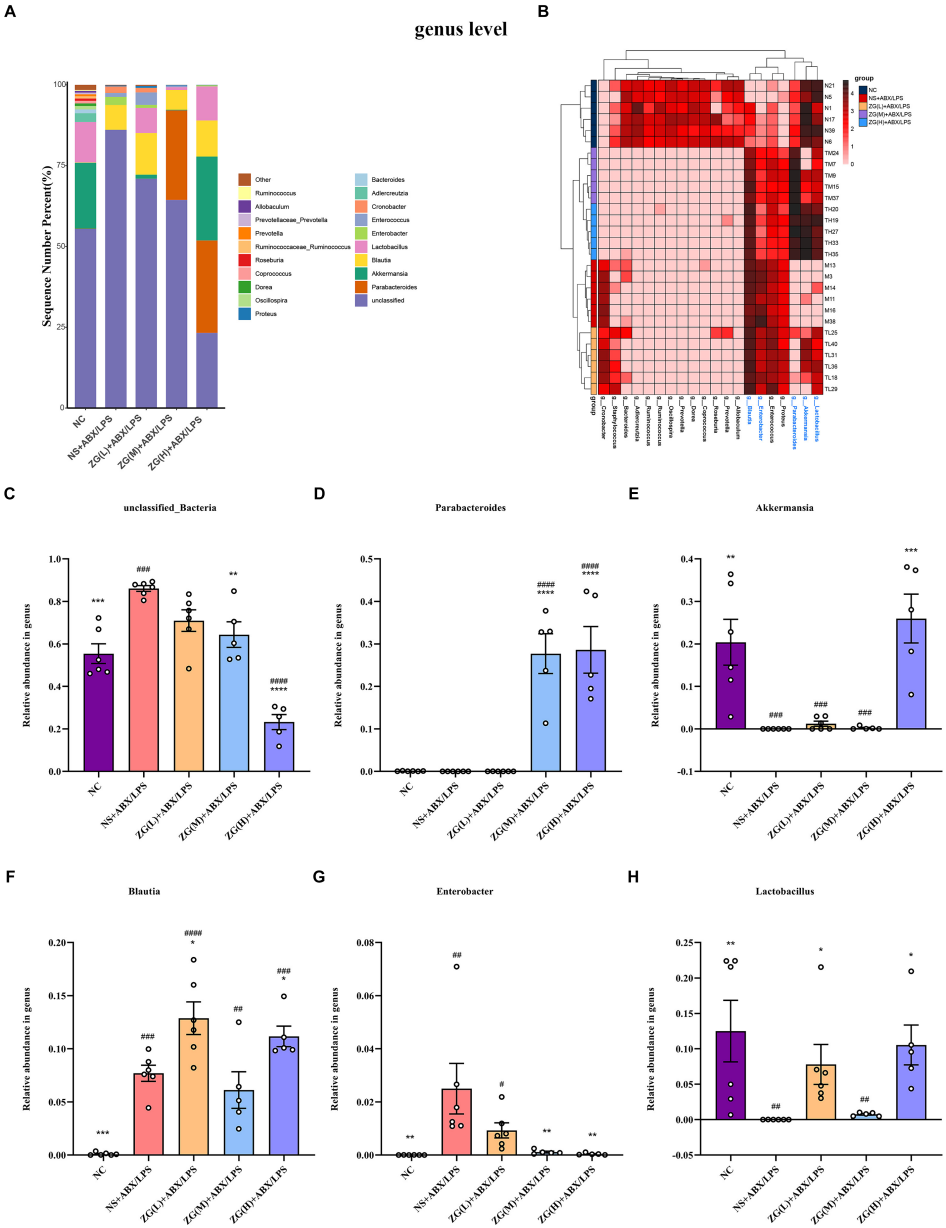
The distribution of cecal content microflora in each group of mice classified at the genus level. **(A)** Histogram of relative abundance ratios. **(B)** Heat map of absolute abundance clustering. **(C–H)** Histogram of relative abundance of dominant bacterial genera. Compared with group NC: ^*^p < 0.05, ^**^p < 0.01, ^***^p < 0.001, ^****^p < 0.0001; Compared with group NS + ABX/LPS: ^#^*p* < 0.05,^##^*p* < 0.01, ^###^*p* < 0.001,^####^*p* < 0.0001.

The samples were clustered based on top 20 species in absolute abundance and then a cluster heat map was constructed to analyze the similarities or differences between different samples or groups. Figures 12B, 13B illustrate that the NC group had independent clusters in the clustering heat map, indicating that its microbial community structure was relatively stable and significantly different from that of other experimental groups. ZG(L) + ABX/LPS and NS + ABX/LPS exhibited similar clustering, which implied that there was some degree of similarity in the microbial community structure between the two. ZG(M) + ABX/LPS and ZG(H) + ABX/LPS also revealed similar clustering, suggesting that the medium- and high-dose ZG treatment groups were comparable in terms of the microbial community structure and that these two groups were closer to each other relative to the other groups. In summary, the cluster heatmap results showed the dose-dependent effect of ZG treatment on the microbial community structure. The microbial community structure in the medium- and high-dose treatment groups exhibited a trend closer to the normal state.

#### 3.8.4 Phylum classification level

To further investigate differences in the dominant microbial flora in the cecal contents of mice in each group, the top 20 species at the phylum level were selected for analysis. Figure 12A shows that 17 phyla were detected in the microflora of mice cecum contents, and the top 5 dominant phyla were *Proteobacteria*, *Firmicutes*, *Bacteroidetes*, *Verrucomicrobia*, and *Actinobacteria*. These five phyla collectively comprised over 98% of the gut bacteria in relative abundance across all samples. Subsequent comparative analyses were conducted on the relative abundance of the dominant phyla among the first four ones (Figures 12C–F). Notably, the NS + ABX/LPS group showed a significant increase in *Proteobacteria* (*p* < 0.0001) and a significant or very significant decrease in *Bacteroidetes*, *Verrucomicrobia*, and *Firmicutes* compared to the NC group (*p* < 0.001 or *p* < 0.0001). Furthermore, *Proteobacteria* were significantly or extremely significantly lower in the ZG(M) + ABX/LPS and ZG(H) + ABX/LPS groups (*p* < 0.001 or *p* < 0.0001). *Bacteroidetes* were significantly higher in the ZG(M) + ABX/LPS and ZG(H) + ABX/LPS groups (*p* < 0.001). *Firmicutes* were significantly higher in the ZG(L) + ABX/LPS and ZG(H) + ABX/LPS groups (*p* < 0.01 or *p* < 0.05). *Verrucomicrobia* were significantly higher in the ZG(H) + ABX/LPS group (*p* < 0.001) compared with the NS + ABX/LPS group.

#### 3.8.5 Genus classification level

To investigate variations in the predominant microbial flora within the cecal contents of mice across each group, we analyzed the top 20 species at the genus level. Based on Figure 13A, 20 bacterial genera were detected in the microbial flora of the mice cecum. The top six dominant bacterial genera were *unclassified_Bacteria*, *Parabacteroides*, *Akkermansia*, *Blautia*, *Enterobacter*, and *Lactobacillus*. These six-genus comprised more than 98% of the gut bacteria in terms of relative abundance in all samples. Further comparative analyses of the relative abundance of the dominant genus of the first 6 were performed, as shown in Figures 13C–H. Compared with NC, *unclassified_Bacteria*, *Blautia*, and *Enterobacter* in NS + ABX/LPS were significantly higher (*p* < 0.001 or *p* < 0.05), *Akkermansia* and *Lactobacillus* were significantly lower (*p* < 0.001 or *p* < 0.05). Compared with NS + ABX/LPS, *Blautia* and *Lactobacillus* were significantly higher in ZG(L) + ABX/LPS and ZG(H) + ABX/LPS groups (*p* < 0.05). *Parabacteroides* were significantly higher in ZG(M) + ABX/LPS and ZG(H) + ABX/LPS groups (*p* < 0.0001). Moreover, *unclassified_Bacteria* and *Enterobacter* were significantly or extremely significantly lower in ZG(M) + ABX/LPS and ZG(H) + ABX/LPS groups (*p* < 0.05 or *p* < 0.0001). *Akkermansia* was significantly higher in the ZG(H) + ABX/LPS group (*p* < 0.001).

#### 3.8.6 LEfSe analysis

**Figure 14:**
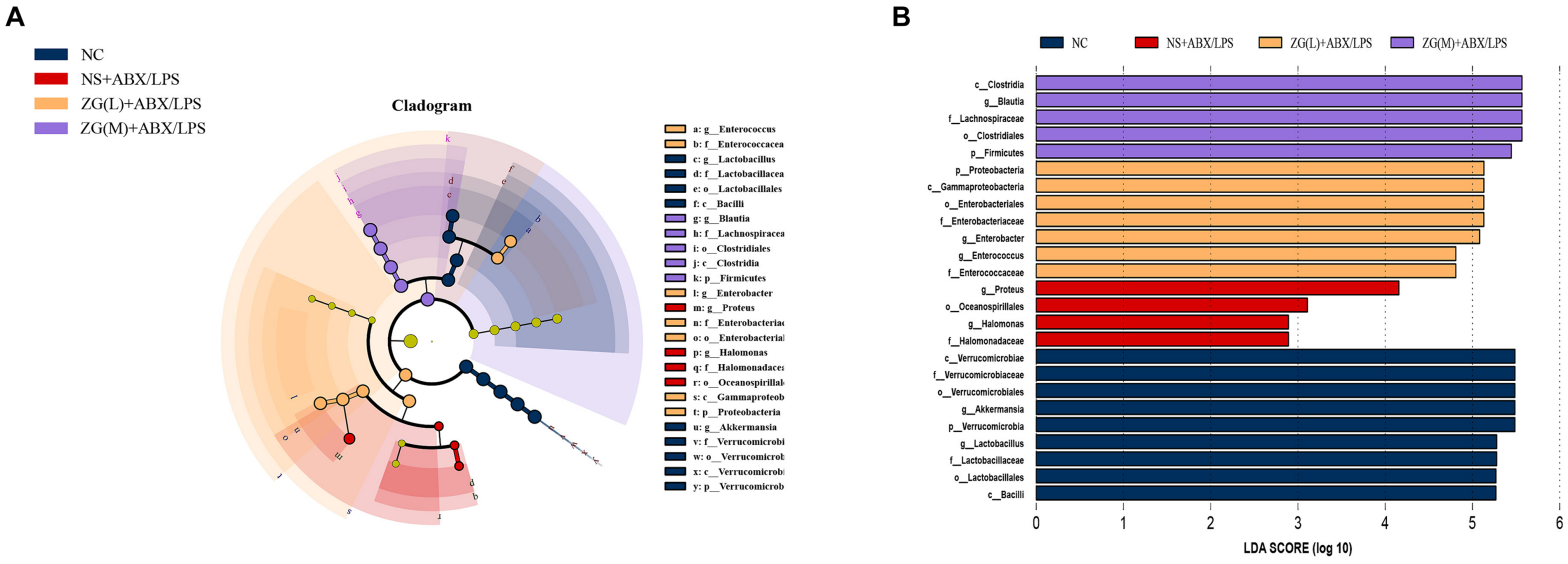
LEfSe analysis of the microflora of the mouse cecum. **(A)** Cladogram. **(B)** Histogram. LDA coefficient cutoff values of 2.

To identify the key biomarkers influencing the microbial variances in the cecal contents of mice, multi-level species difference discrimination (LEfSe) was employed to analyze the various groups at the genus level. The cladogram in Figure 14A illustrates the bacterial taxa which contributed most significantly to microbial differences in the ileal contents of mice in each group. The LDA results in Figure 14B suggest the main bacterial categories with scores exceeding 2 in each group. The bacterial groups with significant differences in NC were *Verrucomicrobia*, *Lactobacillus*, and *Akkermansia*. The bacterial groups with significant differences between NS + ABX/LPS were *Proteus*, *Oceanospirillales*, *Halomonadaceae*, and *Halomonas*. Bacterial groups with significant differences between ZG(L) + ABX/LPS included *Proteobacteria*, *Gammaproteobacteria*, *Enterococcus*, and *Enterobacter*. Bacterial groups with significant differences between ZG(M) + ABX/LPS included *Firmicutes*, *Blautia*, *Clostridiales*, and *Lachnospiraceae*. There were no other species were detected in the ZG(H) + ABX/LPS group.

## 4 Discussion

### 4.1 Effects of zinc gluconate on body weight in mice

The weight of mice in the normal control (NC) group exhibited a consistent upward trend, consistent with typical physiological patterns. However, mice in the NS + ABX/LPS group experienced weight loss as the duration of antibiotic (ABX) usage extended, suggesting that prolonged administration of broad-spectrum antibiotics could result in weight reduction in mice. Exogenous intraperitoneal injection of LPS linearly decreased the weight of the mice, indicating that acute inflammation and intestinal barrier damage induced by LPS adversely affected the overall health of mice. Studies have explored the relationship between inflammation and weight regulation in animal models and human studies. Research by Patterson et al. showed that LPS could destroy the intestinal barrier, causing intestinal microecological components, endotoxins, and other substances to enter the bloodstream, triggering a systemic inflammatory response. In such cases, the mice might have experienced loss of appetite and inadequate energy intake, resulting in weight loss (Patterson et al., 2022). A study by Medeiros et al. showed that zinc supplementation reduced the fecal shedding of *Shigella flexneri* in zinc-deficient mice, improved body weight, and reduced intestinal inflammation (Medeiros et al., 2019). These findings are consistent with those obtained in this study, suggesting that ZG treatment can promote weight gain in mice to a certain extent, especially at high doses. Our results demonstrate the potential of ZG to confer intestinal protection and repair.

### 4.2 Effect of zinc gluconate on mouse feces

It has been shown that zinc has several intestinal health benefits. Zinc is not only a cofactor of various enzymes and regulates cell metabolism, but also maintains and repairs the intestinal barrier (Ohashi and Fukada, 2019; Sarkar et al., 2019). Thus, ZG can potentially alleviate diarrhea symptoms by enhancing the repair of IECs and attenuating the intestinal damage caused by the dual induction of antibiotics and LPS. Our results indicated that high-dose ZG was the most effective in reducing diarrhea scores on the 12th and 14th days of the experiment. These findings are consistent with several studies indicating that higher doses of zinc may improve intestinal mucosal barrier and facilitate the restoration of IECs (Su et al., 2023). Existing literature has highlighted the anti-inflammatory, antioxidant, and epithelial cell repair properties of ZG (DiSilvestro et al., 2015; Bücker et al., 2020). These benefits might be attributed to mechanisms such as the regulation of cell adhesion, stimulation of cell proliferation, and mitigation of inflammatory reactions. Such actions of ZG could contribute to preserving intestinal barrier integrity and impeding the advancement of intestinal diarrhea.

### 4.3 Effects of zinc gluconate on ileum morphology in mice

The morphological structure of the small intestinal mucosa, intestinal injury score, and histopathological grading are often considered important evaluation indicators of the digestion and absorption, intestinal health and functional recovery processes. Among them, the height of small intestinal villi and the depth of crypts are regarded as important indicators of the digestion and absorption function in the small intestine (Ding et al., 2004; Wu et al., 2019). Shortened villus height implies lower surface area for nutrient absorption, while deeper crypt depth indicates faster turnover of new villus cells. In addition, VCR can be a robust marker for assessing intestinal function and health status (Rubin and Levin, 2016). Under light microscopy, analysis of ileal tissues revealed severe damage to the intestinal mucosa in mice subjected to dual antibiotics and LPS induction. The mucosa displayed significant shedding of epithelial cells from the intestinal villi, along with widened intercellular spaces, granulocyte infiltration, and disrupted crypt structures. Moreover, the reductions in villus height, crypt depth, and the VCR were observed, indicating impaired intestinal barrier function in mice. In this study, we found that treatment with different doses of ZG, ZG(L) + ABX/LPS slightly improved the pathological damage of the ileum tissue in mice, but did not completely restore the normal structure. In comparison, ZG(M) + ABX/LPS and ZG(H) + ABX/LPS treatment significantly improved the pathological damage of ileum tissue, as evidence by the reduced mucosal damage, increased intestinal villus height and crypt depth, and increased goblet cells. ZG is known to possess anti-inflammatory and antioxidative properties, which could contribute to its protective effects on the intestinal mucosa. Specifically, ZG has been shown to inhibit the production of pro-inflammatory cytokines and reduce oxidative stress, both of which are implicated in the pathogenesis of intestinal barrier dysfunction and mucosal damage. Additionally, ZG has been reported to modulate immune responses and promote mucosal healing, further supporting its potential in ameliorating intestinal injury. It is important to emphasize how the observed improvements in ileal morphology correlate with the known pharmacological properties of ZG. The observed protective effects of ZG on ileal morphology could be attributed to its multifaceted mechanisms of action. Firstly, ZG possesses anti-inflammatory properties, which may play a pivotal role in mitigating mucosal damage. By inhibiting the production of pro-inflammatory cytokines, such as IL-6 and tumor necrosis factor-alpha (TNF-α), ZG can attenuate the inflammatory response triggered by dual induction of antibiotics and LPS. This anti-inflammatory effect helps to reduce tissue inflammation and prevent further damage to the intestinal mucosa. Secondly, ZG exhibits antioxidative properties, which are crucial for protecting against oxidative stress-induced mucosal injury. Oxidative stress, characterized by an imbalance between ROS production and antioxidant defense mechanisms, contributes to intestinal barrier dysfunction and mucosal damage. ZG acts as a scavenger of ROS and enhances the activity of endogenous antioxidant enzymes, thereby reducing oxidative damage to the intestinal epithelium and promoting mucosal integrity. Furthermore, ZG has been reported to modulate immune responses, including the regulation of immune cell infiltration and cytokine production. By modulating the immune microenvironment in the intestine, ZG can attenuate immune-mediated damage to the mucosa and promote tissue repair and regeneration. Collectively, these results imply that ZG can protect against the damage of intestinal mucosal morphology and structure caused by dual induction of antibiotics and LPS, especially the ZG(H) + ABX/LPS formulation.

### 4.4 Effects of zinc gluconate on the ultrastructure of mouse ileum

Intestinal microvilli and TJ are key structures of IECs which maintain the stability of the intracellular environment, regulating epithelial permeability, and maintaining cell polarity. IECs increase the intestinal surface area by forming finger-like protrusions, known as intestinal microvilli, which promotes the absorption of nutrients and invasion defense against antigens (Jauregi-Miguel, 2021). Under an electron microscope, normal and healthy intestinal microvilli exhibit a columnar, orderly, tightly packed, and evenly arranged appearance. This organized arrangement and consistent density are vital for the proper functioning of IECs and maintaining the stability of the intestinal environment. Therefore, both intestinal microvilli and TJs modulate the IEC function and intestinal health. TEM analysis of the ultrastructure of IECs at the microscopic level has demonstrated that IECs from NC mice exhibit normal morphology. Microvilli are neatly arranged with a tight structure, while TJs, intermediate junctions, and desmosome structures are clearly visible. Intercellular spaces are compact, and the distribution of the nucleus and cytoplasm is even. Moreover, the structures of mitochondria, rough endoplasmic reticulum, and lysosomes appear intact. The IECs of NS + ABX/LPS mice exhibited abnormal morphology, significant shedding of microvilli, structural damage to the TJ, disappearance of the intermediate junction, and desmosomal structures, widening of the intercellular space, dissolution of the epithelial cell membrane, damaged and sunken nucleus, swollen mitochondria, among other alterations. Following ZG treatment, the morphology of IECs in ZG(L) + ABX/LPS and ZG(M) + ABX/LPS mice was partially improved, but some pathological features such as partial loss of microvilli, sparse and disordered arrangement, blurred TJ structure and mitochondrial swelling could still be observed. However, the microvilli of IECs in ZG(H) + ABX/LPS mice were satisfactorily repaired, and the TJ, intermediate junction and desmosome structures could be visualized. This study demonstrates that dual induction with antibiotics and LPS can destroy the morphology and TJ structure of mouse intestinal epithelial microvilli, as well as the mechanical barrier of the mouse intestinal mucosa, thereby increasing intestinal permeability. ZG confers protection against the detrimental effect of dual antibiotics and LPS induction on IECs microvilli and TJ, thereby reducing intestinal mucosal permeability and facilitating the restoration of the compromised intestinal mucosal barrier. Zinc, as a critical micronutrient, modulates cell signal recognition and enhances intestinal barrier function. Zhou et al. demonstrated that exogenous Zn-Asp can mitigate damage induced by DON exposure through the regulation of mouse intestinal stem cell renewal and regeneration capacity, thus ameliorating intestinal morphology and barrier function (Zhou et al., 2020). Furthermore, oral administration of ZnBM has been shown to restore the integrity of LPS-damaged Caco-2 cell monolayers and significantly enhance barrier function (Liu L. et al., 2023).

### 4.5 Effects of zinc gluconate on TJ-related gene and protein expression in mice

IECs are the outermost cells in the intestine. They establish a tight barrier via the TJ and control the permeability of intestinal substances. These TJs contain numerous types of proteins, including structural proteins (ZOs), transmembrane proteins (Occludin and Claudins), and junction adhesion molecules (JAMAS) (Laukoetter et al., 2006; Allaire et al., 2018). The integrity of the TJ complex is crucial in preventing the infiltration of substances like bacteria, pathogens, and endotoxins into the intercellular space. Disruption of intestinal mucosal TJ proteins can elevate intestinal mucosal permeability, resulting in bacterial translocation, infection, diarrhea, and related symptoms (Kayama et al., 2020; Oda et al., 2022). Furthermore, the contractility of IECs significantly impacts intestinal mucosal permeability. Myosin and actin in the cytoskeleton modulate the contraction of IECs and are regulated by MLCK (González-Mariscal et al., 2008; He et al., 2008). It has been demonstrated that myosin function is altered by intestinal inflammation, leading to disruption of the TJ and increased intestinal permeability (Naydenov et al., 2016).

The expression level of ZO-1 influences the integrity of the mucosal barrier and thus the formation of TJs and integrity of epithelial cells. Disruption of ZO-1 may damage the mucosal barrier and increase permeability to harmful substances (Kuo et al., 2021, 2022). Evidence from prior investigations demonstrate that LPS modulates the function and structure of epithelial cells by causing excessive production of intracellular oxidative stress (ROS) and release of pro-inflammatory factors (TNF-α, IL-6), and decreasing the expression of mechanical barrier proteins (ZO-1 and Occludin) (Wu et al., 2018). The findings of our study indicated a significant reduction in ZO-1 expression in the ileal mucosa of mice subjected to both antibiotic and LPS induction. This decline in ZO-1 expression may compromise the normal function of this TJ protein within the intestinal mucosal epithelium, thereby weakening the intestinal mucosal barrier function. Previous research suggests that zinc ions may potentially enhance the integrity and functionality of the mucosal barrier. This could be attributed to zinc’s ability to modulate cell signaling pathways and exert antioxidant and anti-inflammatory effects (Ohashi and Fukada, 2019). In the Caco-2 model, Wang et al. found that zinc altered the morphology of the intestinal TJ, regulated the structure and function of the intestine, and enhanced the intestinal barrier function (Wang et al., 2013). In our study, we also found that ZG promoted the expression of ZO-1 in IECs which strengthened and repaired the intestinal barrier function.

Occludin and Claudins are TJ proteins existing between IECs and are responsible for maintaining the intestinal mucosal barrier and reducing inflammatory extravasation (Da et al., 2022). Hua et al. have showed that the down-regulation of occludin resulted in intestinal barrier impairment. The decrease in occludin expression level is driven by inflammation, antibiotic interventions, and exposure to endotoxins such as LPS, all of which disrupt the TJ among IECs (Hua et al., 2023). The present findings are consistent with this, demonstrating that the combined induction of antibiotics and LPS significantly decreased occludin gene and protein expression in the ileal tissues of mice. Furthermore, previous research suggests that zinc may positively regulate the expression of mucosal barrier proteins to produce inhibit inflammation, oxidative damage and promote epithelial cell repair (Souffriau et al., 2020; Wu et al., 2023). Yi et al. showed that zinc oxide (ZnO) increased the height of ileal villi and enhanced the expression of Occludin in the duodenum and ileum, as well as increased the mRNA expression levels of MUC-2 and ZO-1 in the jejunum and ileum. In this way, it reduced diarrhea symptoms and improved intestinal barrier function in weaned piglets (Yi et al., 2023). These findings are consistent with our results. ZG may repair and maintain the intestinal mucosal barrier by positively regulating Occludin expression. Meanwhile, the regulation of Occludin by ZG exhibits a dose-dependent pattern, with higher doses potentially being more efficacious in promoting intestinal barrier repair. Downregulation of claudin-1 expression decreases the number and complexity of TJs, thereby weakening the barrier function of the intestinal epithelium. Abnormal expression and distribution of claudin-1 results in breakage of TJ chains and formation of holes, allowing ions and macromolecular solutes to enter via the intercellular space, causing damage to the intestine and reducing the barrier function of the intestine (Schmitz et al., 1999; Fischer et al., 2014). Prior research demonstrated that Claudin-1 plays a critical role in maintaining intestinal barrier integrity by regulating paracellular permeability (Saeedi et al., 2015). Furthermore, Atsugi et al. demonstrated that Claudin-1 knockout mice exhibited severe barrier dysfunction, leading to epidermal dehydration and ultimately death, highlighting the essential role of Claudin-1 in mucosal barrier function (Atsugi et al., 2020). Claudin-1 forms the mucosal barrier, and its downregulation has been linked to intestinal inflammation and injury (Zhong et al., 2015; He et al., 2019). Our findings suggest that the combined induction of antibiotics and LPS may have disrupted Claudin-1 expression, thereby impairing the intestinal barrier. Zinc has been shown to positively regulate mucosal barrier integrity and intestinal health. It is plausible that ZG enhances tight junctions by promoting Claudin-1 expression, facilitating the repair of damaged intestinal mucosa. The differential effects of varying doses of ZG on Claudin-1 regulation may be attributed to dose-dependency.

The mRNA expression of JAMA and JAM2 is a commonly used as an indicator for assessing the intestinal barrier integrity (Wong and Kinstler, 2023). It has also been reported that JAM-A can promote the repair of mucosal damage in the JAM-A gene-deficient (JAM-A^−/−^) mouse model of colitis. Overexpression of JAM-A decreases the permeability of IECs, which in turn increases the body’s immune compensation (Vetrano et al., 2008). Our findings indicate that the dual induction of antibiotic- and LPS-induced ileal mucosal damage in mice promoted a significant downregulation of JAM-A mRNA and protein expression levels, which further damaged the intestinal mucosal barrier. ZG treatment significantly increased the expression levels of JAM-A mRNA and protein, suggesting that ZG regulate the repair of intestinal mucosa barrier by regulating JAM-A.

The adhesion between IECs and the stability of the pericellular skeleton are crucial for maintaining the integrity of the intestinal barrier. MLCK is an enzyme that regulates IEC contraction and intercellular junction stability by phosphorylating myosin light chains, thereby influencing intestinal permeability. In response to inflammation or damage, the expression and activity of MLCK increase, resulting in IEC contraction and the loosening of intercellular connections. Furthermore, intestinal injury can compromise the intestinal epithelial barrier, allowing endotoxins like LPS to penetrate. LPS can activate MLCK, thereby damage TJ protein structure of IECs leading to intestinal barrier destruction and increased permeability, making it easier for bacteria, toxins, and other harmful substances to enter the intestinal wall and enter the blood circulation. This triggers inflammatory reactions and diseases (Han et al., 2017; Zhang et al., 2022). Application of the MLCK inhibitor (ML-7) enhanced LPS-induced HPAEC activity, reduced the expression of p-MLCK, and weakened the phosphorylation of myosin light chain or MLCK, thereby stabilizing vascular barrier function (Bai et al., 2009). Elsewhere, MLCK gene knock out or treating wild-type mice with MLCK inhibitors prevented the phosphorylation of MLCK in IECs, maintained TJs structure, reduced protein leakage, and alleviated diarrhea caused by T cell activation (Clayburgh et al., 2005). These findings align with our current investigation, underscoring the importance of MLCK in preserving intestinal barrier integrity and its association with diarrhea pathogenesis. Our study reveals that ZG treatment decreases MLCK expression, suggesting it as one mechanism behind its protective effects. By inhibiting MLCK, ZG may safeguard intestinal barrier function and mitigate inflammatory damage. In summary, our results suggest that ZG can restore intestinal mucosal permeability induced by the dual induction of antibiotics and LPS in mice by up-regulating expression of structural proteins, transmembrane proteins, and adhesion-linkage molecules in the TJ, as well as down-regulating the expression of myosin light-chain kinase.

### 4.6 Effects of ZG on intestinal mucosal permeability in mice

Serological testing of intestinal barrier function may contribute to the diagnosis of intestinal barrier dysfunction. Studies have shown that DAO, D-LA, and ET in serum are the main biomarkers of intestinal mucosal permeability and indirectly reflect intestinal mucosal barrier damage (Yu et al., 2022).

DAO is a highly active structural enzyme expressed in IECs, and its blood levels can be an indicator of intestinal barrier integrity. DAO is usually present in blood circulation in very small amounts, and its levels are influenced by the maturity and integrity of the intestinal mucosa (Camara-Lemarroy et al., 2021). Damage to the intestinal mucosal barrier can lead to a leak of DAO into the bloodstream. This rise in serum DAO levels can be a valuable indicator of the severity of intestinal mucosal diseases (Li et al., 2010). D-LA is a metabolite of intestinal bacteria which may be produced by several types of bacteria in the intestine (such as *Escherichia coli*, lactobacillus, Klebsiella, etc.). Given that no other tissue can produce or metabolize D-LA, the D-LA detected in the blood -LA is only derived from the intestines (Li et al., 2016). Disruption of the intestinal barrier integrity results in an increase in intestinal permeability, allowing the passage of D-LA produced by gut bacteria through the damaged mucosa into the bloodstream. Consequently, blood D-LA level is a potential clinical indicator for assessing intestinal barrier permeability in patients and identifying the translocation of intestinal endotoxins and bacteria post-surgery. ET, a toxic molecule found in the cell walls of Gram-negative bacteria, is released upon bacterial death or rupture (Vindigni et al., 2016). ET is a component of LPS and is commonly present in the cell walls of Enterobacteriaceae. The intestine harbors large amounts of LPS, primarily secreted from intestinal bacteria. Under normal conditions, the intact intestinal barrier prevents LPS from entering the bloodstream. However, following damage of the intestinal barrier function, the permeability of the mucosal barrier is increased, allowing LPS to enter the intestinal mucosa and the bloodstream, resulting in LPSemia. Therefore, monitoring peripheral blood LPS levels serves as a vital approach for assessing bacterial translocation across the intestinal barrier (Earley et al., 2015; Li W. et al., 2023).

In this study, we comprehensively assessed the functional status of the intestinal barrier by evaluating altered intestinal mucosal permeability, the degree of damage to intestinal mucosal cells, and bacterial translocation endotoxemia using the three indices of DAO, D-LA, and ET, respectively. The results indicated that the levels of DAO, D-LA, and ET in the serum of NS + ABX/LPS mice were significantly higher compared with those of NC mice. The serum levels of DAO, D-LA, and ET in various ZG treatment groups were significantly or extremely significantly reduced. These findings indicate that the combined administration of antibiotics and LPS increased intestinal mucosal permeability in mice. However, ZG treatment significantly alleviated the increase in intestinal mucosal permeability induced by the combined use of antibiotics and LPS. Therefore, ZG may protect the intestinal tract from damage by reducing the permeability of the intestinal mucosal barrier and/or improving intestinal barrier integrity. Moreover, there is evidence that *Proteobacteria* can secrete endotoxins. Elsewhere, Earley et al. also reported that downregulation of S24-7 in *Bacteroidetes* promoted burn-induced bacterial translocation and increased intestinal permeability (Wessels et al., 2017). In this study, a correlation was observed between the rise in intestinal mucosal permeability and serum ET levels in model mice, which exhibited decreased *Bacteroidetes* and *Firmicutes* abundance and increased abundance of *Proteobacteria* in the microbial barrier, consistent with previous reports. This suggests that ZG may preserve intestinal barrier integrity by inhibiting dysbiosis induced by dual antibiotics and LPS, thereby reducing endotoxemia. However, further research is needed to elucidate whether ZG maintains intestinal barrier function through microbiota modulation and its underlying mechanism.

### 4.7 Effects of ZG on TLR4/NF-κB signaling pathway in mouse ileum

The TLR4/NF-κB signaling pathway regulates the maintenance and repair of intestinal barrier. Research indicates that activation of this pathway can improve the adhesion of IECs and decrease intestinal permeability, thereby enhancing the integrity of the intestinal barrier. However, prolonged or excessive activation of the TLR4/NF-κB signaling pathway may suppress intestinal inflammation, causing harm and intestinal barrier impairment (Samuelson et al., 2022). In previous studies, LPS increased the permeability of TJs in the gastrointestinal mucosal epithelium and damaged its integrity. This process allowed endotoxins to enter the intestine via paracellular osmosis, causing endotoxin translocation and abnormal expression of cytokines, further exacerbating intestinal inflammation and damage (Zhao et al., 2023). Moreover, our results demonstrate that dual induction by antibiotics and LPS activate the NF-κB signaling pathway via TLR4 resulting in inflammation and immune responses.

Zinc can promote the proliferation and repair of IECs and enhance the repair of intestinal damage. Indeed, zinc deficiency decreased intestinal barrier function (Haase and Rink, 2009). Immune mediators such as TLR ligands can elevate intracellular Zn^2+^ by promoting the expression of ZIP transporter proteins and down-regulating ZnT. The concentration of Zinc is a major regulator of monocytes which recognize bacterial LPS and activate TLR4. A moderate increase in free zinc levels may stimulate LPS-activated monocyte secretion of pro-inflammatory factors (Song et al., 2015; Hu et al., 2023). Song et al. reported that zinc improves the integrity of the intestinal barrier in weaned piglets via the MAPK and TGF-β signaling pathways (Lee et al., 2018). Previous research has demonstrated that inhibiting the intestinal NF-κB using zinc-based drugs can lower the levels of cytokines TNF-α and IL-6, thus protecting the intestinal mucosal barrier from damage induced by LPS in young mice (Xu et al., 2018; Xiao et al., 2019). The findings of our study also suggest that ZG may exert its anti-inflammatory properties and maintain the intestinal barrier by modulating the TLR4/NF-κB signaling pathway. However, our study did not explore the specific regulatory mechanism of ZG in the TLR4/NF-κB/p65 signaling pathway and its impact on inflammatory signaling and intestinal barrier function. Further investigations should primarily elucidate these aspects.

### 4.8 Effects of zinc gluconate on microbial diversity in cecal contents of mice

In this study, Illumina NovaSeq sequencing of the 16S rRNA V3 + V4 hypervariable region was employed to assess the composition and alterations of the cecal microbiota in normal mice, mice with induced intestinal mucosal barrier injury through dual antibiotic and LPS administration, and ZG-treated mice. Analysis of Shannon, Simpson, and Observed features indices indicated higher microbial diversity in normal mice compared to model mice, suggesting that the dual induction of antibiotics and LPS reduces intestinal flora diversity. However, each ZG treatment group exhibited varying degrees of increase in the Shannon index and decrease in the Simpson index. These findings demonstrate that ZG treatment contributes to the restoration of bacterial flora diversity and richness. Using the weighted UniFrac algorithm, we compared the composition of the microbiota of in mouse cecum based on the PCoA and NMDS methods. Analysis of scatter plots of PCoA and NMDS scores showed that bacterial flora separation occurred at the ASV level in the normal control group, model control group, and ZG treatment group, indicating that treatment and dosage factors can affect bacterial flora composition. There appears to be a partial overlap in bacterial communities between the NS + ABX/LPS and ZG(L) + ABX/LPS groups, although they are separate from the NC, ZG(M) + ABX/LPS, and ZG(H) + ABX/LPS groups. This suggests that low-dose ZG treatment may have limited restorative effects on the intestinal flora. In addition, there is partial overlap between the ZG(M) + ABX/LPS and ZG(H) + ABX/LPS groups, with some similarity observed with the NC group, indicating potential similarities in bacterial flora composition among the mid- and high-dose ZG treatment groups. The recovery of bacterial flora in the intestinal tract may offer some beneficial effects. Overall, we hypothesize that the therapeutic dosage of ZG may exhibit a threshold effect on modulating the composition of the intestinal flora.

Furthermore, we analyzed the distribution of intestinal microbial colony content among groups at the phylum and genus levels. In this study, *Proteobacteria*, *Firmicutes*, *Bacteroidetes*, *Verrucomicrobia*, and *Actinobacteria* were the dominant groups, which is consistent with the findings of previous studies (Kim et al., 2022). However, treatment of mice with a double blow of antibiotics and *Escherichia coli* (*E. coli* O55:B5) altered these dominant bacterial groups, with the relative abundance of *Proteobacteria*, *Blautia*, and *Enterobacter* significantly increased. The relative abundance of *Firmicutes*, *Bacteroidetes*, *Verrucomicrobia*, *Lactobacillus*, and *Akkermansia* was significantly reduced. These findings align with the anticipated dysbiosis of microbial communities induced by the combined use of antibiotics and LPS.

*Proteobacteria* are the main pathogenic bacterial phylum which contributes to symptoms such as intestinal infections, diarrhea, immune imbalance, and dysbiosis. Research shows that *Proteobacteria* is the main bacterial phylum in the intestine, and its high abundance may indicate a biological disorder in the microflora (Huang et al., 2019). *Enterobacter* is a facultative anaerobic bacterium. The excessive proliferation of *Enterobacter* can trigger an abnormal activation of the host’s immune system, resulting in an inflammatory response. Consequently, it is commonly viewed as a signaling indicator of pathogenic microorganisms. In this study, the relative abundance of *Proteobacteria* and *Enterobacter* in the NS + ABX/LPS group was significantly increased suggesting that dual induction of antibiotics and LPS caused imbalance in the intestinal flora of mice. However, ZG(M) + ABX/LPS and ZG(H) + ABX/LPS treatment significantly reduced the abundance of *Proteobacteria* and *Enterobacter*. These results indicate that ZG intervention may have a counter-regulatory effect on the relative abundance of *Proteobacteria* and *Enterobacter*.

*Firmicutes* are integral to intestinal health, contributing significantly to the digestion of complex carbohydrates by producing enzymes and generating beneficial metabolites like short-chain fatty acids (such as propionic, acetic, and butyric acids). These compounds not only supply energy to intestinal cells but also support the integrity of the intestinal mucosal barrier. Moreover, *Firmicutes* play a crucial role in inhibiting the proliferation and infiltration of pathogenic bacteria through various mechanisms, including the production of antimicrobial substances, regulation of intestinal pH, and reinforcement of the intestinal mucosal barrier. As a result, *Firmicutes* protect the intestinal tract from potential infections and maintain its overall well-being (Kapoor et al., 2023). Although glycosides cannot be digested by the body into short-chain fatty acids, *Bacteroidetes* can degrade them thereby providing calories and nutrients to the body. High abundance of *Bacteroidetes* can therefore improve the function of the intestinal mucosal barrier and enhance the innate immune response. A study by Sonnenburg J.L. showed that *Bacteroidetes* can inhibit bacterial growth by prompting IECs to secrete substantial mucus and binding it to peptidoglycan on the bacterial surface. Moreover, *Verrucomicrobia*, a group of microorganisms often found in the intestinal mucus layer, produce metabolites that stimulate IECs, exerting a regulatory influence in the intestinal environment. In addition, *Verrucomicrobia* can activate the immune response via activating the G protein-coupled receptors (Kim et al., 2021). *Lactobacillus* belongs to the group of *Firmicutes* and is considered a probiotic. It produces lactic acid which inhibits the colonization and growth of pathogenic bacteria in the intestinal mucosal epithelium by decreasing intestinal pH. Furthermore, lactic acid bacteria can stimulate the secretion of adhesins, prevent the apoptosis of IECs, and consequently, help uphold the stability of the intestinal mucosal barrier (Miranda et al., 2018; Choi et al., 2020). *Akkermansia* is a potentially beneficial gut bacterium classified as *Verrucomicrobia*. It can increase the thickness of the intestinal mucosa, break down the mucus on the intestinal surface, and suppress the production of inflammatory cytokines, thereby improve the intestinal barrier function and stability of the intestinal microbiota (Derrien et al., 2017; Plovier et al., 2017). In our investigation, the NS + ABX/LPS group exhibited a significant decrease in the relative abundance of *Bacteroidetes*, *Verrucomicrobia*, *Firmicutes*, *Parabacteroides*, *Lactobacillus*, and *Akkermansia*. This suggests that the combined induction of antibiotics and LPS disrupted the balance of intestinal flora in mice. Specifically, *Bacteroides* and *Parabacteroides* showed significant increases in the ZG(M) + ABX/LPS and ZG(H) + ABX/LPS groups, while *Firmicutes* and *Lactobacillus* were significantly increased in the ZG(L) + ABX/LPS and ZG(H) + ABX/LPS groups. *Verrucomicrobia* and *Akkermansia* were significantly increased in ZG(H) + ABX/LPS. Altogether, our results suggest that ZG can restore the relative abundance of the above bacteria, but the performance varied among different treatment groups, suggesting a ZG dose-dependent effect.

## 5 Conclusion

In this study, we investigated the mechanism of action of ZG in AAC. The experimental results showed that ZG significantly reversed the adverse outcomes such as weight loss, fecal abnormalities, morphological and structural disruption of the ileal mucosa, increased permeability of the intestinal barrier, and disruption of the microbiota in mice treated with the dual induction of antibiotics and LPS. In terms of mechanism of action, it regulated the expression of TJ-related genes, inhibited the TLR4/NF-κB signaling pathway, modulated serum levels of DAO, D-LA, and ET, and adjusted microbial community structure. These findings provide new perspectives for understanding the mechanisms of intestinal mucosal barrier maintenance and the treatment of related diseases.

Multiple factors were introduced into the intestinal mucosal injury model established using antibiotics combined with LPS to more closely match the actual disease situation. Meanwhile, for the first time, we investigated the potential of ZG to repair intestinal mucosal barrier damage in mice administered with dual-induced antibiotics and LPS, and its impact on intestinal flora. Further in-depth is advocated to explore the molecular mechanisms of ZG action, especially via the TLR4/NF-κB signaling pathway. Few studies have explored the time window of application and dose of ZG in the treatment of diseases. Further investigations should systematically evaluate the influence of these factors on therapeutic outcomes. Although this study primarily focused on *in vivo* animal experiments, no experimental validation was conducted *in vitro* or through clinical study. Therefore, additional research is warranted to validate the feasibility and efficacy of these findings in human subjects.

## Data availability statement

16S rRNA data presented in the study are deposited in the NGDC BioProject repository, accession number PRJCA024397.

## Ethics statement

The animal study was approved by The Experimental Care and Ethics Committee of Guangxi Medical University. The study was conducted in accordance with the local legislation and institutional requirements.

## Author contributions

YW: Conceptualization, Data curation, Investigation, Methodology, Software, Visualization, Writing – original draft, Writing – review & editing. JX: Data curation, Investigation, Methodology, Software, Writing – review & editing. SW: Methodology, Writing – review & editing. YS: Investigation, Writing – review & editing. XY: Investigation, Writing – review & editing. SS: Investigation, Writing – review & editing. LL: Supervision, Writing – review & editing. XC: Supervision, Writing – review & editing. TH: Supervision, Writing – review & editing. QS: Data curation, Resources, Supervision, Writing – review & editing.
