# Supplementary material for: Protective effect of zinc gluconate on intestinal mucosal barrier injury in antibiotics and LPS-induced mice
Source: Front Microbiol. 2024 May 23;15:1407091. doi: 10.3389/fmicb.2024.1407091 (PMC11157515; doi:10.3389/fmicb.2024.1407091)
Supplement: Supplementary file 1 [file Table_1.DOCX]

**Table S1. Genes and primer sequences used for RT-PCR**

| **Gene** | **Primer sequence（5′-3′）** | **Product size(bp)** | **Accession number** |
| --- | --- | --- | --- |
| GAPDH | F:AGAAGGTGGTGAAGCAGGCATC | 111 | NM_001411843.1 |
|  | R:CGAAGGTGGAAGAGTGGGAGTTG |  |  |
| ZO-1 | F: ACCCGAAACTGATGCTGTGGATAG | 105 | XM_036152895.1 |
|  | R:GCTGGCTGGCTGTACTGTGAG |  |  |
| Occludin | F: CACACCTCGTCGCTAGTGC | 128 | NM_001360536.1 |
|  | R:CTCCCAAGATAAGCGAACCTGC |  |  |
| Claudin-1 | F:GTGTCCTACTTTCCTGCTCCTGTC | 80 | NM_016674.4 |
|  | R:AGAAGGTGTTGGCTTGGGATAAGG |  |  |
| JAMA | F: ACCTACTCTGGCTTCTCCTCTCC | 117 | NM_172647.2 |
|  | R:GAAGGTGACTCGGTCCGCATAG |  |  |
| MLCK | F:GACTACGACGAAGACGGCAACTG | 184 | NM_001408263.1 |
|  | R:CCTCCTCCTCCTCCTCCTCCTC |  |  |

**Table S2. Composition of the electrophoretic adhesive configuration system**

| **Gel concentration** | **Ingredients** | **Volumetric (ml)** |
| --- | --- | --- |
| 12%Gel |  | 15 |
|  | H_2_O | 4.9 |
|  | 30%Acr/bis | 6.0 |
|  | 1.5Mtris-HCl（pH8.8） | 3.8 |
|  | 10% SDS | 0.15 |
|  | 10%APS | 0.15 |
|  | TEMED | 0.006 |
| 10% Gel |  | 15 |
|  | H_2_O | 5.9 |
|  | 30%Acr/bis | 5.0 |
|  | 1.5Mtris-HCl（pH8.8） | 3.8 |
|  | 10% SDS | 0.15 |
|  | 10%APS | 0.15 |
|  | TEMED | 0.006 |
| 8% Gel |  | 15 |
|  | H_2_O | 6.9 |
|  | 30%Acr/bis | 4.0 |
|  | 1.5Mtris-HCl（pH8.8） | 3.8 |
|  | 10% SDS | 0.15 |
|  | 10%APS | 0.15 |
|  | TEMED | 0.009 |
| 5% Gel |  | 6 |
|  | H_2_O | 4.1 |
|  | 30%Acr/bis | 1.0 |
|  | 1.0Mtris-HCl（Ph6.8） | 0.75 |
|  | 10% SDS | 0.06 |
|  | 10%APS | 0.06 |
|  | TEMED | 0.006 |

**Table S3. WB-related gene transfer condition**

| **Gene name** | **Transmembrane current** | **Transmembrane time** |
| --- | --- | --- |
| β-actin, ZO-1 | 300mA | 150min |
| β-actin, Occludin | 200mA | 120min |
| β-actin, Claudin-1 | 200mA | 120min |
| β-actin, TLR4 | 200mA | 120min |
| β-actin, NF-κB/p65 | 200mA | 120min |

**TableS4. Comparison of changes in body weight of mice across groups（x±s）**

| **Date** | **NC** | **NS+ABX/LPS** | **ZG(L)+ABX/LPS** | **ZG(M)+ABX/LPS** | **ZG(H)+ABX/LPS** | **F** | ***P*** |
| --- | --- | --- | --- | --- | --- | --- | --- |
| Day 0 | 21.2±0.4 | 21.1±1.1 | 21.4±0.9 | 20.9±0.9 | 21.1±0.6 | 0.29 | 0.884 |
| Day 4 | 22.5±0.6 | 21.2±1 | 21.9±0.9 | 21.1±0.9 | 21.6±0.8 | 2.62 | 0.059 |
| Day 8 | 23.2±0.7 | 21.6±0.5* | 22.3±1 | 21.4±0.9 | 22±0.8 | 4.32 | 0.009 |
| Day 9 | 23.4±0.7 | 18.7±0.7**** | 19.1±0.7 | 18.9±1.1 | 19.1±0.5 | 40.69 | ＜0.0001 |
| Day 10 | 23.4±0.7 | 19.1±0.8 | 19.6±1 | 18.9±0.7 | 19.5±0.8 | 32.65 | ＜0.0001 |
| Day 12 | 23.6±0.8 | 20.2±0.7 | 21±1.4 | 20.7±0.9 | 21.4±0.9## | 12.69 | ＜0.0001 |
| Day 14 | 24.6±0.8 | 20.8±0.6 | 21.7±1.2 | 22±0.9 | 23.5±0.8### | 16.93 | ＜0.0001 |

Note: Compared with group NC: ^*^*P*＜0.05，^****^*P*＜0.0001；Compared with group NS+ABX/LPS: ^##^*P*＜0.01, ^###^*P*＜0.001.
